# Nanostructured porous graphene and its composites for energy storage applications

**DOI:** 10.1186/s40580-017-0123-0

**Published:** 2017-10-30

**Authors:** Pablo Ramos Ferrer, Annsley Mace, Samantha N. Thomas, Ju-Won Jeon

**Affiliations:** Department of Chemical & Biological Engineering, University of Alabama, Tuscaloosa, AL 35487 USA

**Keywords:** Graphene, Porous graphene, Energy storage, Batteries, Self-assembly

## Abstract

Graphene, 2D atomic-layer of sp^2^ carbon, has attracted a great deal of interest for use in solar cells, LEDs, electronic skin, touchscreens, energy storage devices, and microelectronics. This is due to excellent properties of graphene, such as a high theoretical surface area, electrical conductivity, and mechanical strength. The fundamental structure of graphene is also manipulatable, allowing for the formation of an even more extraordinary material, porous graphene. Porous graphene structures can be categorized as microporous, mesoporous, or macroporous depending on the pore size, all with their own unique advantages. These characteristics of graphene, which are further explained in this paper, may be the key to greatly improving a wide range of applications in energy storage systems.

## Introduction

Graphene is defined as a single-layer of sp^2^ carbon structure, which forms two-dimensional hexagonal honeycomb lattice [1, 2]. In 2004 Novoselov and Geim et al. demonstrated single layer graphene by mechanical exfoliation of graphite using scotch tape for the first time [3]. Since this discovery of graphene, it has attracted great attention in scientific communities because of its fascinating properties.

Graphene possesses excellent mechanical strength with a Young’s Modulus of about 1000 GPa [4, 5]. Effectively, it has been used to enhance the strength and stability of other materials [4, 5]. Even though graphene displays characteristics of a strong, conducting metal, it also has the capabilities of a manipulatable, flexible structure, making it a good candidate for flexible electronics [4, 5]. Additionally, graphene has a high thermal conductivity, of between 2000 and 5000 W/m K [6–10]. This allows it to be especially useful in thermal management in various applications [6–10]. It also has remarkable optical properties. One of which is that a large portion of incident white light (~ 2.3%) can be absorbed by a single-layer graphene sheet, which makes it an attractive prospect for energy harvesting applications [11–16].

**Fig. 1:**
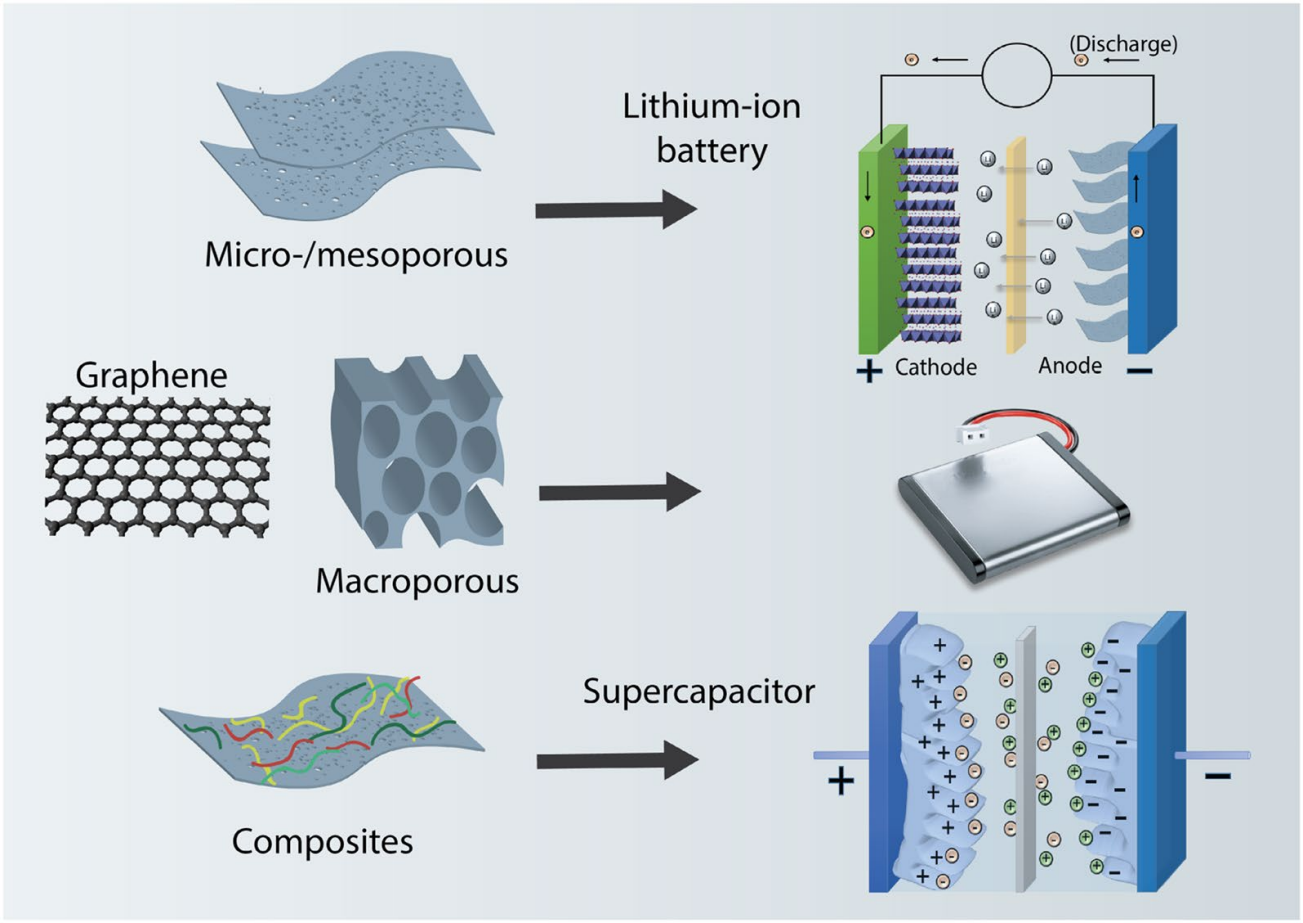
Schematic representation of graphene-based materials and their applications in energy storage

Graphene sheet can be further assembled or processed into porous structures that exhibit desired physical and chemical properties, Fig. 1. Porous graphene is a modified graphene with pores on the sheet and/or between the sheets that, as a result, has unique structural and electrochemical properties that are different from pure graphene. Energy storage is a very attractive application for porous graphene due to the increased surface area and additional porosity, which could potentially lead to improved electrochemical performance. Therefore, porous graphene has been extensively studied for various energy storage systems including lithium-ion batteries (LIBs), supercapacitors, lithium–sulfur (Li–S), lithium–air (Li–air), and fuel cells [17–21]. For example, porous graphene could show superior electrochemical properties when used as anode in place of conventional graphite because of its higher surface area [17]. Similarly, electrochemical capacitors, also known as supercapacitors, have also taken advantage of the large specific surface area of porous graphene, proven by several recent studies [22, 23]. A great deal of efforts have been devoted utilizing porous graphene for other advanced energy storage systems such as Li–S and Li–air batteries by fully exploiting its enhanced surface area and porosity [19, 20].

There are several review papers regarding graphene and its application in energy storage [24–29]. However, there are just few review articles on porous graphene and their energy storage applications despite rapid growth in this field [30, 31]. In this review article, we summarize various processing techniques to fabricate nanostructured porous graphene depending on the pore size with an emphasis on energy storage applications. Synthesis of graphene-based porous nanocomposites and their applications in energy storage are also discussed. We expect that this paper will give broad ideas about synthesis, processing, and properties of porous graphene and its composites.

## Porous graphene

Graphene can be produced in a variety of ways including a scotch tape method, chemical vapor depositions (CVD), and chemical oxidations/exfoliation/reduction of graphite using Hummers’ methods [3, 32–37]. Each method has its own advantages and challenges. Using mechanical exfoliation, on one hand, it is difficult to obtain large quantities of graphene, but the small yield of product is of a high quality [3]. CVD could also produce high quality graphene, but it requires specially designed instruments and well-controlled synthesis conditions [32, 38, 39]. On contrary, chemical oxidation/exfoliation/reduction methods could yield a large amount of graphene although resulting graphene, which can also be called reduced graphene oxide (RGO), could have defective sites and functional groups on graphene sheets [37]. Even though electrical conductivity of RGO is lower than others, chemical production of graphene based upon Hummers’ method followed by reduction is particularly suitable for mass production of nanostructured graphene because graphene oxide (GO) is a good basic building block to create a wide range of porous structures using bottom-up approaches [37, 40–42].

Porous graphene can be synthesized using either template or template-free methods. The template approach uses some predetermined structures as the “template” to create the appropriate size and shape of the pores required [43–45]. Alternatively, template free-methods require chemical etching or some other means to introduce modifications onto the surface of the graphene [40, 46, 47]. By carefully controlling assembly parameters and synthesis conditions, pore size and surface area of resulting graphene could be controlled and optimized for specific applications.

Nanostructured porous graphene could be classified as microporous (smaller than 2 nm), mesoporous (2–50 nm), and macroporous (bigger than 50 nm) graphene depending on the pore size according to the International Union of Pure and Applied Chemistry (IUPAC) definition [48]. While in many cases GO is used as the same intermediate and building blocks, it is the ability to alter the microscopic structure of the final structure of graphene that makes this product so widely applicable in energy storage.

### Production of porous graphene

#### Self-assembled macroporous graphene

**Fig. 2:**
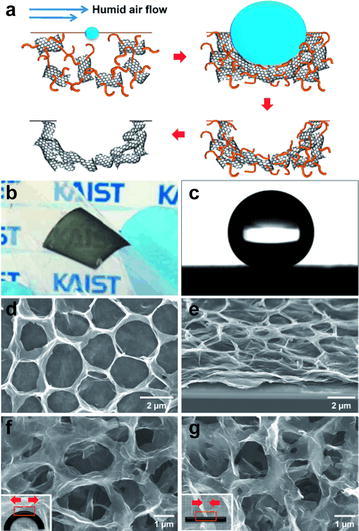
**a** Diagram illustrating the assembly of modified GO into the carbon films using breath-figure method. **b** A photograph of a flexible semi-transparent macroporous RGO film on PET. **c** A water drop on macroporous RGO film. **d**, **e**, **f**, and **g** Plane-view and tilted SEM images of an RGO film (Reprinted with permission [40])

The “breath figure” method is a promising approach to create the three-dimensional macroporous graphene structures possessing relatively large micrometer size pores [17, 40]. The breath figure process allows for finely tuned adjustments of the ordered hexagonal structures by varying solvents, substrates, temperature, humidity, airflow, and other factors [49]. This self-assembly method consists of the following basic steps. First, modification of GO should be performed to make it more hydrophobic because the breath figure method requires GO to be dispersed in an organic phase. A modified GO dispersed in an organic solvent is placed dropwise onto a substrate as humid air flows over [17, 40]. As the temperature of the interface between the air and solution is reduced due to endothermic evaporation of an organic solvent, water condenses into droplets [17, 40]. Because of capillary forces and coagulation, the droplets line up in an ordered way [17, 40, 50]. Eventually, the water droplets evaporate and all that remain are the porous structures in the substrate, which in this case is modified GO sheets, Fig. 2 [40]. This self-assembled modified GO macroporous structure can be reduced by various reduction methods to obtain macropous graphene architectures.

Kim successfully demonstrated flexible macroporous graphene film with controllable pore size ranging from 1 to 4 um using the breath-figure method [40]. In this method, polystyrene-grafted GO was synthesized by surface-initiated atom transfer radical polymerization (ATRP) to make it dispersible in an organic solvent, benzene [40]. This dispersion was drop-cast onto substrates under humid air flow, resulting in flexible macroporous modified GO films. This method demonstrated that the pore size could be controlled by simply altering the concentration of the precursor solution along with the polymer chain lengths on the surfaces of the GO sheet. By increasing the concentration of polystyrene-grafted GO dispersion, they were able to decrease the pore size from 4 to 1 μm [40]. Eventually, macroporous graphene was prepared after heat treatment of self-assembled GO at 1000 °C with hydrogen gas [40]. This macroporous RGO film was used as a supercapcitor electrode, exhibiting 86.7 F/g at a scan rate of 100 mV/s in 1 M H_2_SO_4_ aqueous electrolyte [40]. The capacitance could be further increased to 103.2 F/g after nitrogen-doping through thermal treatment with ammonia gas because of the enhanced conductivity and additional pseudocapacitance from nitrogen heteroatom [40].

**Fig. 3:**
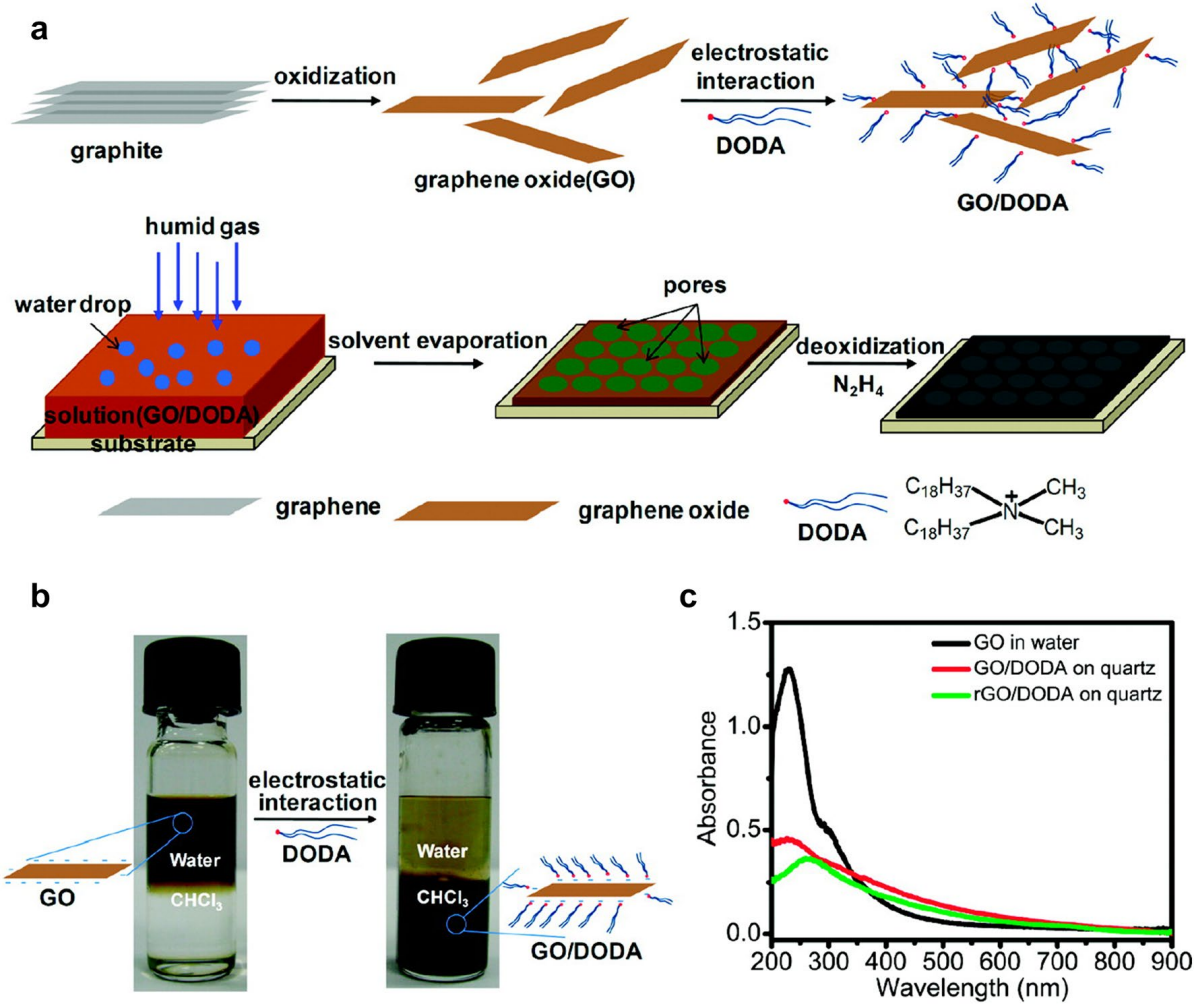
Processes to form honeycomb structures composed of graphene sheets: **a** schematic drawing of the preparation of the honeycomb structured film based on graphene oxide; **b** photographs of the GO solution during phase transfer process by DODA; **c** the UVvis spectra of the GO in water (black), GO/DODA casting film (red), and the rGO/DODA film after exposure to N2H4 vapor (green) (Reprinted with permission [17])

Chen also developed a scalable and facile self-assembly of graphene to fabricate macroporous graphene by the breath figure method [17]. Instead of using complicated polymerization steps for synthesizing modified GO, they adopted low-cost and green approach to modifying graphene oxide to be dispersed in an organic phase using a cationic surfactant, dimethyldioctadecylammonium bromide (DODA·Br), Fig. 3 [17]. This cationic surfactant would self-assemble on the negative GO based on electrostatic interactions, allowing it to be more hydrophobic and be dispersed in chloroform due to the long alkyl chains of DODA [17]. The resulting GO/DODA chloroform dispersion was drop cast on to a substrate under 85% relative humidity [17]. After evaporation of the solvent and water, the 2 μm thick honeycomb film was formed with pore sizes of about 1.5 μm [17]. Following a chemical reduction step with hydrazine vapor, macroporous graphene was obtained [17]. The films exhibited an electrical conductivity of 4.6 S/m and were thermally stable up to temperatures of 400 °C [17]. The high surface area of the disordered honeycomb sheets allowed rapid diffusion of lithium ions, high capacity, and easy access for an electrolyte. Thus, these structures have great potential as anodes for lithium-ion batteries [17]. Using coin cells with Li counter electrode, it showed an initial large specific capacity of 3025 mAh/g and a reversible capacity of 1612 mAh/g. For the first 25 cycles, the reversible capacity was 1300 mAh/g, and after 50 cycles it decreased to 1150 mAh/g [17].

A 3D architecture of macroporous graphene can also be created by in situ self-assembly induced by a simple chemical reduction of GO [46, 47]. This method has great advantages over other reported methods in terms of simplicity, scalability and environmental benignity. Generally, other production methods of macroporous graphene often require sacrificial templates, multiple steps, or precisely controlled assembly conditions [17, 40, 43]. However, with this method, no chemical or physical cross-linkers were used, and assembly can be performed at mild temperature and low pressure [47]. When GO went through chemical reduction by NaHSO_3_, Na_2_S, Vitamin C, HI and hydroquinone without stirring, 3D macroporous graphene hydrogel was generated without using any template or cross-linker [47]. As-prepared hydrogels demonstrated notable mechanical properties. The elastic modulus of the graphene hydrogel was about 0.13 MPa and the yield stress was 28 kPa. Such characteristics are analogous to a chemically cross-linked polymer [47]. The specific capacities of graphene hydrogel were 166 F/g at 10 mV/s and 156 F/g at 20 mV/s [47]. Depending upon the shape of the reactor, the shape of the graphene hydrogel can also be manipulated, providing more flexibility to production [47]. This macroporous graphene hydrogel can be converted to macroporous aerogel via freeze-drying [47]. The graphene aerogel with a density of 15 mg/cm^3^ exhibited a bulk conductivity of 87 S/m, which was higher than crosslinked 3D graphene [47].

In situ self-assembly by chemical reduction was also employed by Wang and coworkers [46]. In this case, by using a different reducing medium consisting of oxalic acid and sodium iodide, 3D macroporous graphene architectures were created even from very diluted GO dispersion of 0.1 mg/ml [46]. Interestingly, the pore size, bulk density and electrical conductivity of graphene aerogels were greatly affected by initial GO concentration [46]. With higher GO concentration, the average pore size of graphene became smaller, and electrical conductivity became higher. For instance, the average pore size of graphene aerogels was 2.1 um when reduced by 4.5 mg/ml GO dispersion, whereas 0.1 mg/ml GO dispersion formed very large pore around 24.2 μm [46].

#### Micro- and mesoporous graphene synthesized by chemical etching

For graphene, it is generally hard to obtain high surface area close to the theoretical value (2630 m^2^/g) because of aggregating nature of graphene sheets caused by strong van der Waals interaction [18, 51]. This restacking of graphene sheets reduces its accessible surface area that can be utilized for energy storage applications [52, 53]. Chemical etching is an effective way to produce micro- and/or mesoporous graphene nanostructures possessing high surface area [22]. The chemical activation method that was previously used to create activated carbons resulted in successful preparation of porous graphene having small pores less than 10 nm [22].

Micro- and mesoporous graphene sheets having extremely high surface area up to 3100 m^2^/g was prepared by simple activation of GO with KOH [22]. The GO/KOH mixture was heated at 800 °C in an inert Ar gas environment [22]. This simple activation process generated nanoscale pores according to the following reaction: 6KOH + C < − > 2 K + 3H_2_ + 2K_2_CO_3_ [22]. It was shown that the activation process etched the microwave treated-GO and generated a three dimensional distribution of meso-and micro pores ranging from 0.3 to 10 nm with a large pore volume of 2.14 cm^3^/g [22]. When its surface area was calculated by the Brunauer–Emmett–Teller method from nitrogen adsorption/desorption measurement, extremely high surface area of 3100 m^2^/g was obtained, which is much higher than the theoretical surface area of defect-free graphene sheets [22].

Micro- and mesoporous graphene, so called holey graphene, has also been prepared using other etching agents including H_2_O_2_, HNO_3_, Fe_2_O_3_ and NaI [23, 54–57]. For example, holey graphene was synthesize through hydrothermal reaction using H_2_O_2_ as a pore forming agent [23]. Beginning with the well-dispersed GO, H_2_O_2_ was added, then the mixture was sealed and heated to 180 °C in a Teflon-lined autoclave [23]. Simultaneously, during this process GO sheets self-assembled into a 3-D framework, while the H_2_O_2_ partially oxidized graphene oxide sheets, leaving vacancies that turn into nanopores [23]. This 3D hydrogel was further reduced in 1 M sodium ascorbate aqueous solution at 100 °C [23]. Freeze-dried holey graphene framework fabricated showed relatively high surface area of 830 m^2^/g, which was higher than non-holey graphene counterpart (~ 260 m^2^/g) [23]. In this process, controlling the amount of the etching agent, H_2_O_2_ is important for successful creation of holey graphene. It was shown that excessive addition of H_2_O_2_ causes aggressive etching that would break the graphene sheets into small pieces [23]. The compressed holely graphene frameworks exhibited excellent supercapacitor performance in both aqueous and non-aqueous electrolytes. In 1-ethyl-3-methylimidazolium tetrafluoroborate/aceonitrile electrolyte, it showed a capacity of 298 F/g, leading to a high gravimetric energy density of 127 Wh/kg [23].

More research explored synthesis of holey graphene oxide using H_2_O_2_ to produce solution processable holey graphene oxide. In this case an aqueous mixture of GO and H_2_O_2_ are heated to 100 °C for 4 h while stirring [58]. After removal of the H_2_O_2_ the resulting HGO can be re-dispersed to form a stable aqueous dispersion. Via a reduction induced self-assembly process, holey graphene hydrogels were obtained from the holey graphene oxide. The resulting hydrogels displayed an interconnected three dimensional porous network that can be formed in many shapes and sizes simply by changing the reactor used in the reduction process. Similarly, this holey graphene possessed improved electrochemical performance because of highly accessible surface area and pores.

Heteroatom doping of graphene is one of the most effective way to enhance its electrochemical performance for energy storage applications because of its modified properties such as additional pseudocapacitance, improved electrical conductivity and surface wettability [40, 56, 59, 60]. Therefore, heteroatom doped holey graphene was demonstrated to further improve its energy storage performance [60–63]. For instance, nitrogen-doped holey graphene was synthesized in combination of chemical etching and hydrothermal treatment [61, 62]. The resulting nitrogen-doped holely graphene retained meso-and macropores providing channels for rapid ion transport within the electrodes while providing enhanced electrical conductivity because of the nitrogen doping [61, 62]. When used as a catalyst for the oxygen reduction reaction (ORR), it showed better better electrocatalytic activity than undoped graphene [61, 62].

### Preparation of nanostructured graphene-based porous composites

Apart from porous graphene and heteroatom-doped porous graphene, graphene-based composite materials could be an ideal platform for energy storage owing to tunable composition-dependent properties. In addition, graphene composites having specifically designed properties such as electrochemical properties, surface area, electrical and thermal conductivities can be synthesized by varying controlling composition and synthesis conditions in line with specific applications. Nanoarchitectures of graphene-based composites could lead to superior energy storage capacities due to their additional surface area as well as larger pore volumes, which could facilitate diffusion of electrolytes when the composites are used as electrodes. So far, a wide range of graphene-based composites have been demonstrated with various organic and inorganic materials through template-assisted synthesis, aerosol spray drying process as well as assembly of graphene with other nanomaterials [18, 43, 51, 52, 64–69].

#### Template-assisted synthesis of nanostructured porous graphene composites

Nanostructured graphene-based hybrid materials can be prepared with the use of a variety of soft and hard templates including polymers, carbon spheres, inorganic particles, micelles, ionic liquids, and nanostructured substrates [18, 43, 44, 51, 70–76]. Template approach is particularly useful to fabricate nanostructured composites having specifically designed porous morphology and pore size because morphology and architecture of resulting composites are determined by templates used during the preparation steps [43, 73]. A diverse range of graphene-based composites including inorganic/graphene, carbon/graphene, and conducting polymer/graphene have been reported via template-assisted synthesis methods [18, 43, 51].

**Fig. 4:**
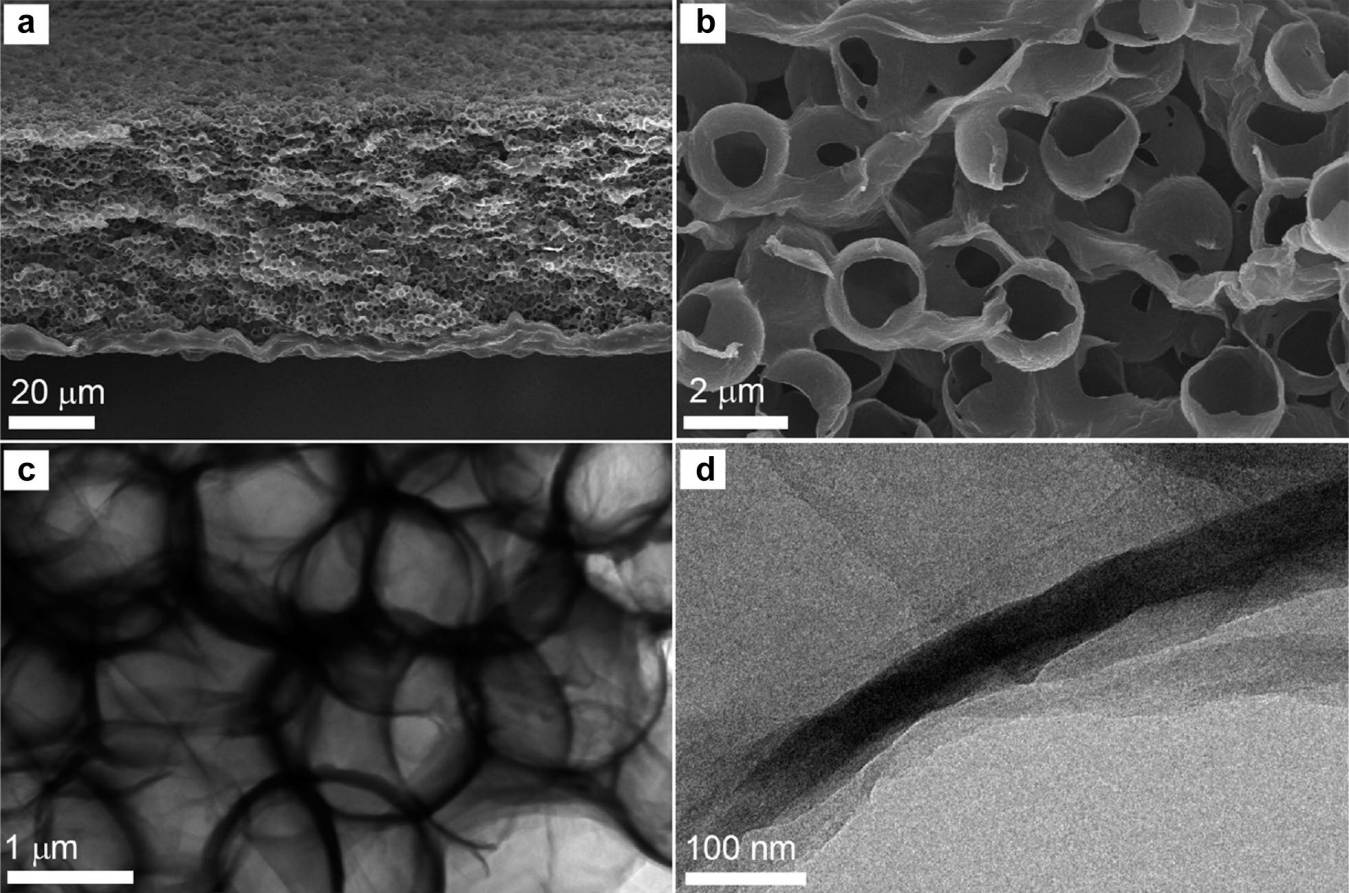
**a** Low-magnified and **b** high-magnified cross-sectional SEM images of the macroporous chemically modified graphene film. **c**, **d** TEM images of the macroporous chemically modified graphene film (Reprinted with permission [43])

For example, three-dimensional macroporous graphene/MnO_2_ composite was successfully prepared through vacuum filtration using a sacrificial polystyrene bead template [43]. In this method, chemically modified graphene (CMG) was mixed with polystyrene (PS) beads, and vacuum filtrated under controlled pH conditions to prevent agglomeration and form continuous composite films [43]. After eliminating PS out of composites, macroporous three-dimensional graphene architecture possessing about 2 μm pores was obtained, Fig. 4 [43]. Afterwards, MnO_2_ was chemically incorporated into this macroporous graphene to increase its electroactivity [43]. This macroporous MnO_2_-graphene composite had significantly increased capacitance (389 F/g at 1 A/g) as compared to the nonporous planar MnO_2_-graphene counterpart (137 F/g) because of higher surface area and facilitated ion transport within three-dimensional interconnected macropores [43]. An asymmetric supercapacitor consisting of macroporous MnO_2_/CMG positive electrode and macroporous CMG negative electrode can be operated in a relatively wide voltage range up to 2 V in NaSO_4_ aqueous electrolyte [43].

A three-dimensional hierarchical carbon nanostructure composed of graphene sheets and mesoporous carbon spheres was also prepared using mesoporous silica spheres (MSS) as a sacrificial template [51]. The approach to synthesizing this three-dimensional carbon nanostructures was similar to the case of aforementioned macroporous to CMG except for the fact that a different type of template and additional post-treatments are used [43, 51]. First, positively charged amino-functionalized MSS colloidal particles were added to negatively charged GO dispersion at pH 3 to form uniformly MSS/GO composites [51]. Mesoporous carbon spheres (MCS) were formed in MSS/GO composites and GO was reduced to RGO when a CVD method was employed at high temperature using ferrocene as a carbon source [51]. Final hierarchical carbon nanostructures consisting of graphene sheets and mesoporous carbon spheres were achieved after removing MSS templates with HF solution [51]. This hierarchical carbon showed substantially larger surface area of 1496 m^2^/g than the RGO counterpart having 801 m^2^/g [51]. After thermal annealing the electrical conductivity of the composite was increased to 381 S/m because of the decreased oxygen-containing functional group. Even though RGO has higher specific capacitance of 218 F/g than the three-dimensional hierarchical carbon composite mainly due to additional oxygen-containing functional groups attached in RGO sheets, this composites showed greater rate capability and power density originating from hierarchical porous structure as well as improved electrical conductivity [51]. These results signify that oxygen-containing functional groups offer additional pseudocapacitance, enhancing its electrochemical performance, whereas electrical conductivity and porous structure of electrodes are also substantially advantageous for supercapacitor performance at high rates.

Hierarchical porous graphene/polyaniline composites are other examples of a successful template-assisted synthesis [18]. In this case, when CaCl_2_ was added in GO dispersion, CaCO_3_ particles were formed on GO sheets, in which CaCO_3_ were used as template to create a macroporous structure [18]. After chemical reduction with hydrazine vapor and removing template with dilute acid, three dimensional interconnected macroporous RGO film was obtained [18]. This macroporous 3-D RGO exhibited increased rate performance and specific capacitance of around 115 F/g at 0.5 A/g in comparison with nonporous RGO counterpart showing about 90 F/g. This is mainly due to higher effective specific surface area and interconnected 3D porous structure, leading to facilitated ion transport [18]. The electrochemical performance was further increased by performing polymerization of aniline on 3-D RGO films [18]. This resulting 3-D RGO/polyaniline (PANI) electrode possessed significantly improved capacitance up to 385 F/g at 0.5 A/g because of high pseudocapacitance contribution of PANI based upon its redox reactions [18]. Moreover, a flexible supercapacitor was demonstrated using macroporous RGO/PANI electrodes, showing great flexibility and stable performance [18].

#### Crumpled graphene-based nanocomposites

**Fig. 5:**
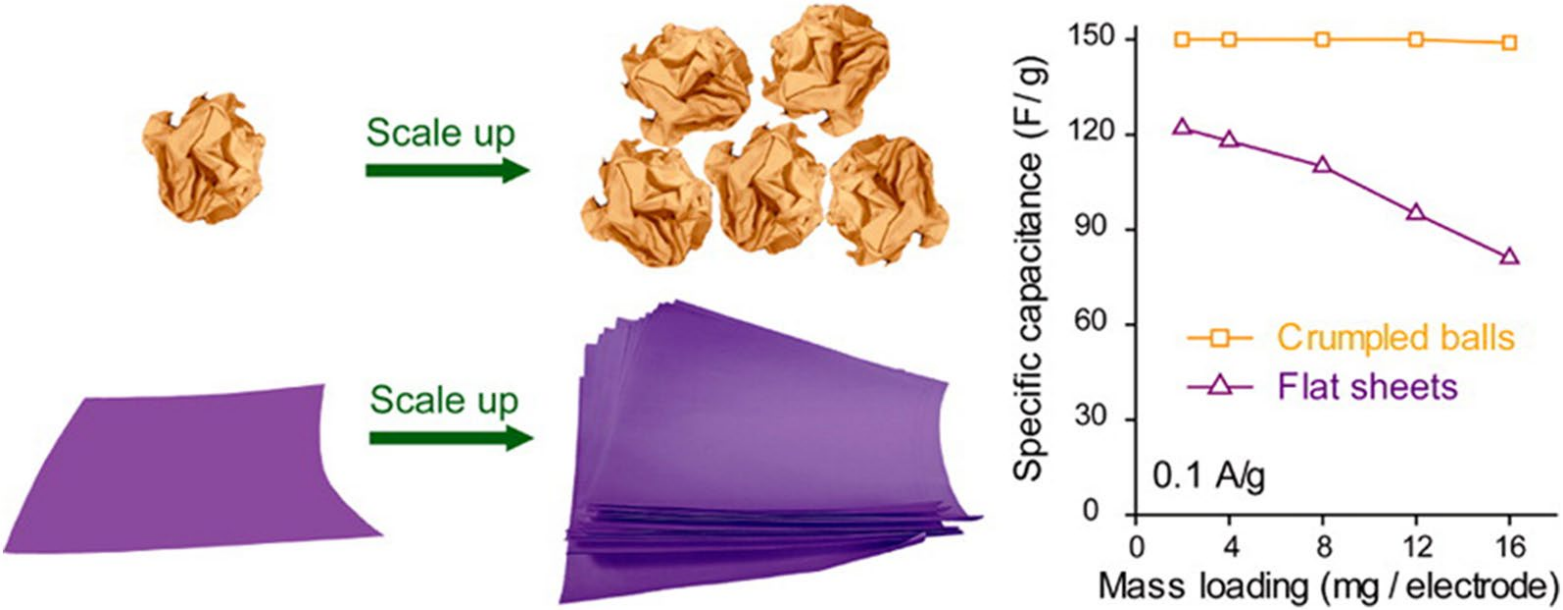
Schematic illustration of crumpled graphene balls and uncrumpled graphene sheets with their specific capacitances depending on the electrode mass loading (Reprinted with permission [53])

Graphene sheets has a tendency to restack and form lamellar structure because of strong van der Waals force between graphene sheets [53, 77]. The aggregation of graphene sheets reduce the accessible surface area, often resulting in inferior electrochemical performance [53, 77]. In order to prevent undesirable restacking and aggregation of graphene sheets, crumpled ball-like graphene was demonstrated [53, 77]. Crumpled ball-like graphene nanostructure was successfully synthesized via an aerosol spray drying process followed by chemical or thermal reduction [53, 77]. When micrometer-sized droplets of GO precursors generated by ultrasonic atomizer passed through preheated furnace, the droplets were shrunk by rapid evaporation because of capillary compression, leading to crumpled ball-like nanostrcutures [53, 77]. The crumpled graphene balls showed substantially improved electrochemical performance in terms of capacitance and rate capability due to its higher surface area and uniformly distributed pores [53, 77]. Notably, capacitances of crumpled graphene balls were not largely affected by the electrode mass loading while uncrumpled flat graphene sheets showed mass-loading-dependent capacitance, Fig. 5.

This development opened up the new opportunities for synthesizing crumpled graphene-based ball-like nanocomposites for energy storage applications [52, 64–68]. The same groups, Jang and Huang employed this aerosol-assisted assembly to synthesize Si nanoparticles encapsulated with crumpled graphene sheets for lithium-ion battery anode [64]. The mixture Si and GO was nebulized, and assembled in preheated furnace at high temperature, 600 °C and further reduced at 700 °C [64]. This unique nanostructures could mitigate typical issues of Si electrode such as low cycling stability, low Coulombic efficiency, and excessive formation of a solid electrolyte interphase (SEI) layer. When used as an anode, crumpled graphene sheets could accommodated volume expansion of Si during lithiation process, leading to improved cycling stability and Coulombic efficiency of Si/RGO balls due to flexibility of graphene shells [64]. In addition to Si/RGO, Pt-nanoparticles/RGO nanostructured was also successfully produced in a similar manner, which showed promising electroactalytic activity for a methanol oxidation reaction [52, 65].

Other crumpled graphene nanocomposites were also reported. For instance, a redox-active crumpled graphene/few-walled carbon nanotubes electrode was created using the partially reduced crumpled graphene oxide and functionalized few-walled carbon nanotubes mixture through vacuum-filtration process [78]. It was shown that this hierarchical nanostructure can exhibit improved capacities of up to ~ 170 mAh/g and rate capability compared to geometrically different counterpart with a similar composition. The high capacity and cycling stability can be attributed to fast ion diffusion and electron transport as well as redox-active oxygen-containing functional groups attached on partially reduced graphene oxide sheets and carbon nanotubes [78].

The aerosol-spray process was further extended to fabricate 3-D crumpled graphene/carbon nanotube/polyaniline (CCP) nanocomposites for supercapacitor applications [66]. First, GO/carbon nanotube (CNT)/PANI tertiary dispersion was formed after the polymerization of aniline was carried out in the mixture of GO and functionalized CNT [66]. Following the aerosol-spraying process, crumpled ball-like nanocomposite was obtained. Instead of conventional inactive and insulating polyvinylidene fluoride (PVDF) binder, graphene sheets were used as a binder to hold the materials together and form interconnected 3D structures. In this CCP nanocomposites, crumpled graphene and CNT could prevent restacking of graphene sheets while PANI provided additional pseudocapacitance based on its redox activity. This nanocomposites showed high capacitance up to 456 F/g when tested in KOH aqueous electrolyte [66].

#### Layer-by-Layer (LbL) Assembly for porous graphene-based nanocomposites

Layer-by-layer (LbL) assembly is a pervasive film fabrication technique that is characterized by its formation through alternating deposition of complementary species [79–83]. Multilayer films are created based upon electrostatic interactions, hydrogen boding, and/or other interactions between complementary species [79–84]. LbL assembly is especially versatile due to its simplicity and control that can be exhibited with this technique. Different film properties can be achieved by changing the conditions at which the films are assembled [80, 85, 86]. LbL assembly can be carried out in various manners such as dip-, spray-, spin- and vacuum-assisted methods [67, 68, 87–89]. In recent years LbL assembly has received great attention from many fields of research due to the great potential it has in film technologies [79–83].

With the demand for greater energy from micro-power sources, LbL assembly assists in pioneering a new era in micro-batteries or micro-capacitors for thin film devices [90–95]. Porous nanostructured graphene-base composites can be constructed by LbL assembly when assembled with other nanostructured materials [67, 95, 96]. For example, all carbon multi-walled carbon nanotubes/chemically reduced graphene oxide (MWCNT/CRG) electrode was fabricated through LbL assembly [95]. One-dimensional MWCNTs provide void volume in the resulting LbL films, and no additional binder is required. First, MWCNTs were functionalized with amine groups to render them positively charged, and negatively charged GO dispersion was directly used for LbL assembly [95]. The MWCNT/GO LbL films were chemically reduced to MWCNT/CRG using hydrazine vapor in order to increase electrical conductivity [95]. The capacity of 160 F/cm^3^ was obtained in an acidic electrolyte originating from electric double layer capacitance and redox activity of oxygen-containing functional groups [95].

**Fig. 6:**
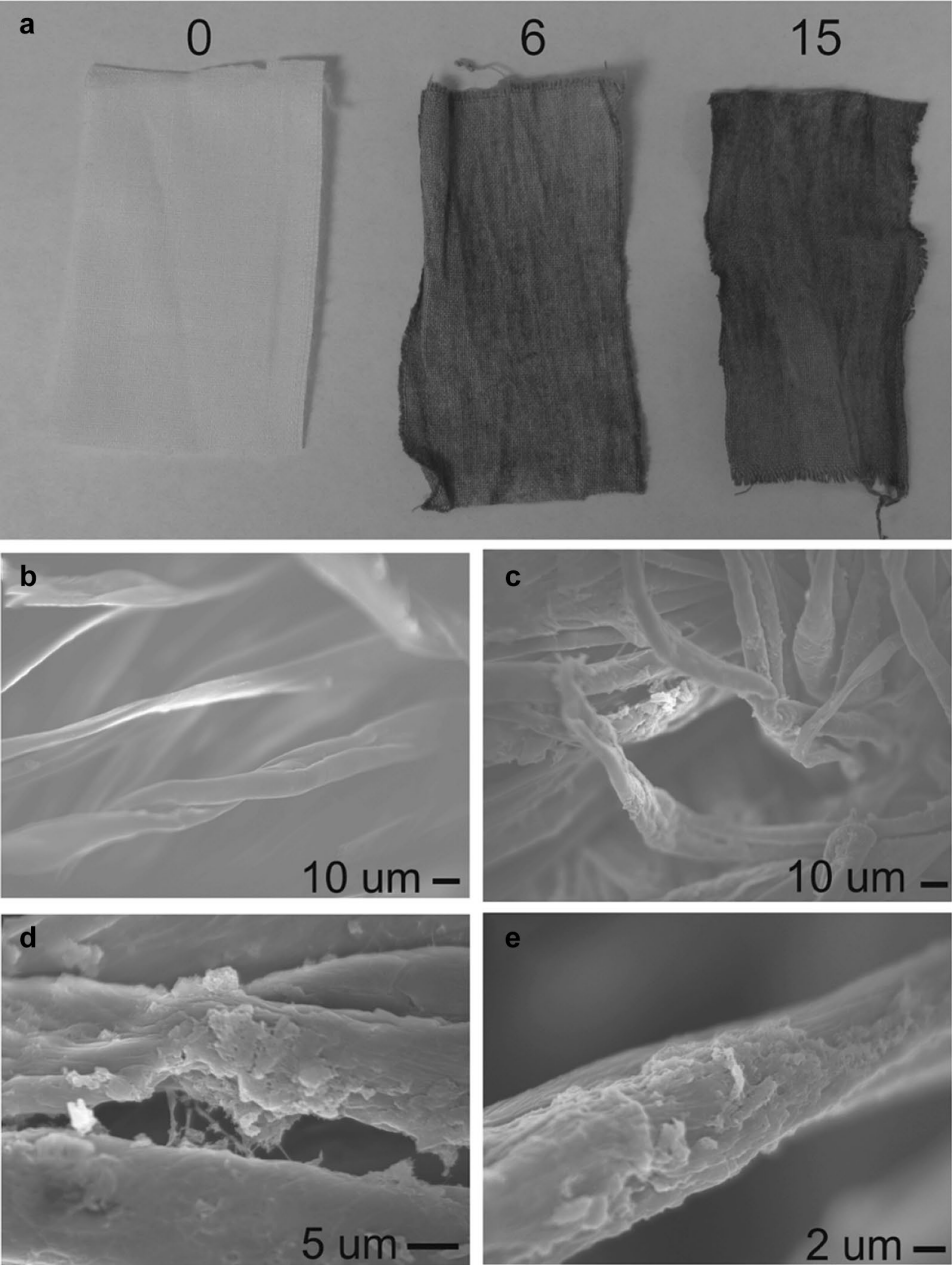
**a** Digital image of PANI NF/GO LbL films on cotton fabric, from left to right (0, 6, and 15 layer pairs). SEM images of **b** bare cotton fabric and **c**–**e** 15 layer pairs of PANI NF/GO on cotton fabric (Reprinted with permission [67])

Porous polyaniline nanofiber/reduced graphene oxide (PANI NF/RGO) hybrid films were also demonstrated using dip-assisted LbL assembly followed by electrochemical reduction [67]. The LbL assembly was performed with positively charged PANI NFs and negatively charged GO dispersion [67]. The film growth was affected by pH values of the GO dispersion; lower pH of GO dispersion led to thicker film growth presumably due to the degree of ionization of oxygen-containing functional groups of GO [67]. The LbL films were also successfully deposited onto a nonplanar complex substrate, cotton fabric, Fig. 6 [67]. The void fraction of LbL film calculated was 0.625, indicating 62.5% of LbL film are porous, which can facilitate ion transport during charge/discharge processes [67]. In spite of high porosity, electrochemical performance was greatly affected by film thickness owing to ion transport limitation in thicker electrodes [67]. The 1520 nm thick PANI NF/RGO film showed 184 mAh/cm^3^ at 0.03 A/g while the 1520 nm thick film exhibited 123 mAh/cm^3^ [67].

Spray-assisted LbL assembly was adopted to fabricate the compositionally similar electrodes, PANI NF/RGO and address the previously encountered issues of dip-assisted LbL assembly such as slow processing, cross contamination, and scalability problem [96]. With this form of LbL assembly, the parameters on the graphene-based electrodes were mostly affected by the rinsing and blow-drying times [96]. With fine tuning of those parameters, the optimal result could be obtained by eliminating the rinsing step and adding intermediate-blow drying [96]. The resulting films had a growth rate that was much higher than its dip-assisted counterpart [67, 96]. The electrodes also had a lower density and higher porosity (74% void) [96]. This highly porous nature resulted in improved rate capability as well higher power density [96].

For flexible power systems, nanostructured aramid nanofibers (ANFs)/graphene sheets multilayer films were fabricated from ANFs and GO dispersions using dip-assisted LbL assembly [69]. In this case, because both ANFs and GO are negatively charged, electrostatic interaction between ANFs and GO was not a driving force for the LbL assembly. Instead, hydrogen bonding and van der Waals interactions are the main driving force for the film assembly [69]. After reduction of GO, its electrochemical activity increased due to recovered sp^2^-hybridized graphitic domains. Notably, These LbL films showed excellent mechanical flexibility and durability when subjected to repeated flexure, indicating that this ANFs/graphene LbL films can be used in structural energy systems.

## Energy storage using porous graphene and graphene-based nanocomposites

Energy storage is becoming more important to meet ever-increasing energy demands. To improve current energy storage efficiency, a wide range of materials have been explored as an electrode for energy storage devices. Among various materials, graphene is drawing great interest due to its excellent properties including good mechanical strength, high thermal and electrical conductivities, good chemical stability, and high theoretical surface area [2, 7, 51, 97]. Nanostructured porous graphene is particularly beneficial for energy storage applications because of its higher surface to volume ratio and larger pore volume. In the next sections, we will introduce researches on porous graphene and graphene-based porous composites in energy storage applications including lithium-ion batteries, supercapacitors, lithium-sulfur batteries, and lithium-air batteries.

### Supercapacitors

Supercapacitors, also called ultracapacitors, can have higher power density than batteries, which means that supercapacitors can deliver energy much faster (within a few seconds) than batteriers [98]. Supercapacitors are also capable of storing 100–1000 of times more energy than conventional electrolytic capacitors [98]. However, energy densities of supercapacitors (1–10 Wh/kg) are much lower than batteries (100–150 Wh/kg) [98, 99]. Supercapacitors can be classified electric double layer capacitors (EDLCs) and pseudocapacitors depending upon their charge storage mechanisms [100, 101]. EDLCs store charges based upon the electrostatic adsorption of electrolyte ions on porous carbon electrodes while pseudocapacitors utilize redox reactions of electroactive species such as conducting polymers and metal oxides [100, 102].

**Fig. 7:**
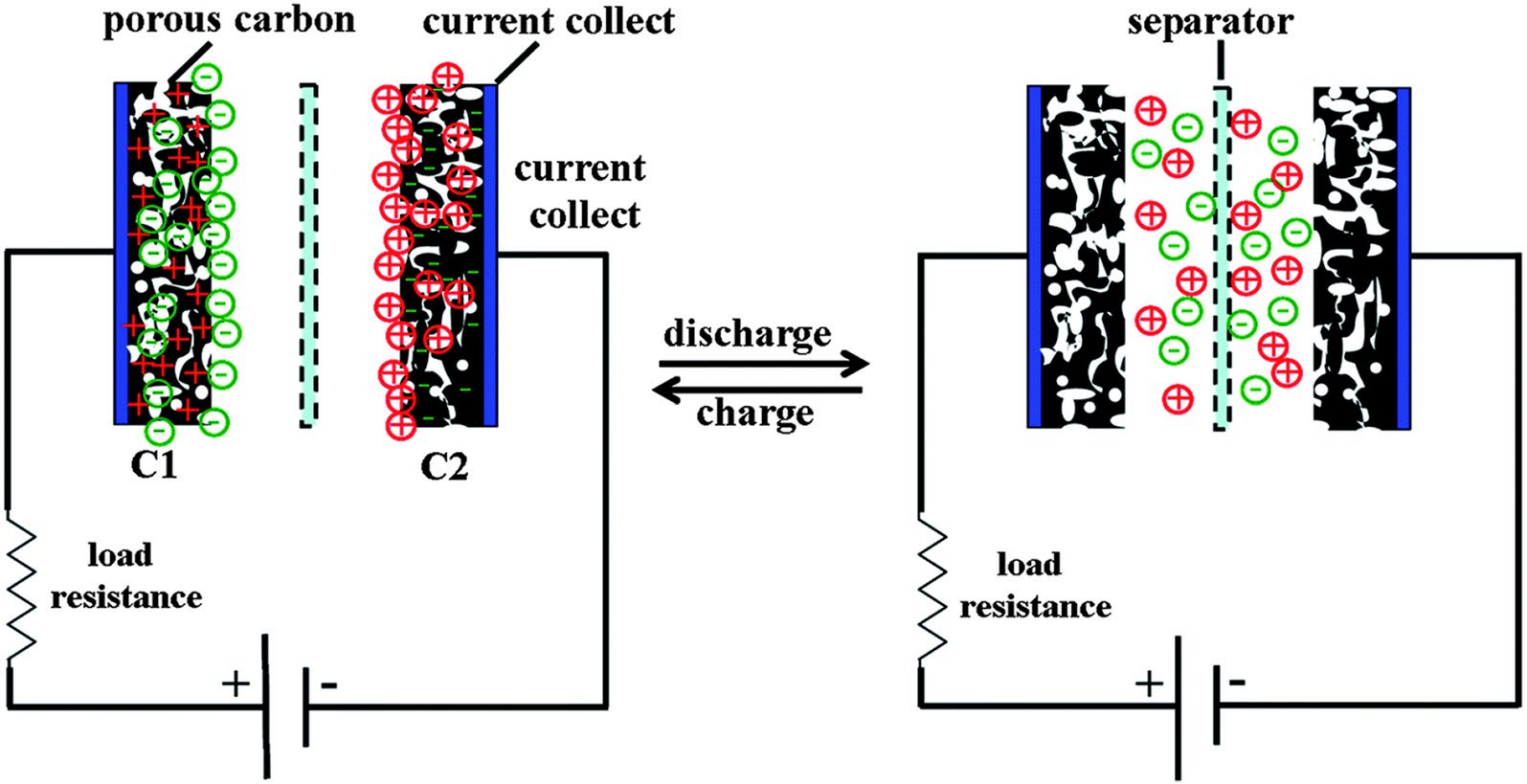
Schematic diagram of the charged and discharged electric double layer capacitor (Reprinted with permission [101])

Capacitor performance of EDLCs can be evaluated based on capacitance, which is the electrostatic charge accumulation at the interface of the electrode/electrolyte, Fig. 7. For EDLCs, capacitance can be described based on the following Equation [98, 103, 104]

1 $$C = \frac{{\varepsilon_{r} \varepsilon_{0} A}}{d}$$

where ɛ_r_ is the electrolyte dielectric constant, ɛ_0_ is the dielectric constant of vacuum, d is the effective thickness of the electric double layer, and A is the effective surface area of the electrode [98, 103, 104]. As the Eq. (1) suggested, capacitance is directly proportional to the effective surface are of the electrode, which implies that electrodes possessing higher surface area could lead to higher capacitance as well as higher energy density. It should be noted that the effective surface area is a surface area that is accessible by electrolytes, not necessarily the same as the one calculated by nitrogen adsorption/desorption measurements. Porous graphene and porous graphene-based hybrids have been extensively studied as electrode materials for supercapacitors because of their high theoretical surface area along with good mechanical strength and stability.

#### Macroporous graphene for supercapacitors

Macroporous graphene structures generally possess larger pore and pore volume as compared to micro- and mesoporous graphene. Electrolytes can be held within large macropores, which could reduce required diffusion path length of electrolyte ions. Indeed, macroporous self-assembled graphene hydrogels prepared by one-step hydrothermal process exhibited relatively high a specific capacitance of 152 F/g at a scan rate of 20 mV/s in 5 M KOH aqueous electrolyte [105]. When 3D macroporous graphene was created by in situ self-assembly induced by chemical reduction of GO, it displayed a capacity of 156 F/g at 20 mV/s [47]. These capacities of macroporous graphene hydrogels were higher than that of nonporous planar graphene (101 F/g) measured under the same conditions [106]. The increased electrochemical performance of macroporous graphene can be attributed to rapid ion transport due to the porous structures [47, 105]. However, 3D macroporous graphene prepared by the breath figure method showed rather lower capacities (87 F/g for pyrolyzed macroporous RGO, and 103 F/g for N-doped macroporous RGO) in H_2_SO_4_ electrolyte comparing with nonporous graphene electrodes [40]. These results indicate that macroporous structure does not necessarily guarantee higher capacities because there are many other factors affecting supercapacitor performance such as electrical conductivity, electrolytes, and surface properties [100, 102, 103, 107, 108].

#### Holey graphene for supercapacitors

Micro- and mesoporous graphene, also called holey graphene, is a particularly promising for supercapacitor applications because extremely high surface area can be obtained in these materials [22]. For supercapacitor applications, high surface area can provide more active sites for electric double layer capacitive mechanism, which could lead to higher supercapacitor performance [98]. For instance, activated graphene by KOH having surface area of 3100 m^2^/g showed promising electrochemical performance in a non-aqueous electrolyte [22]. In 1-butyl-3-methylimidazolium tetrafluoroborate/acetonitrile (BMIMBF_4_/AN) electrolyte, a capacitance of 166 F/g was measured in the voltage window of 3.5 V [22]. The energy density was found to be ~ 70 Wh/kg, which is around four times higher than existing activated carbon-based supercapacitors [22]. The power density of ~ 75 kW/kg found for the synthesized packaged cell was around an order of magnitude higher than commercial supercapacitors carbon of ~ 4–5 Wh/kg [22]. When holey graphene sheets etched by H_2_O_2_ was assembled into 3D frameworks and formed hierarchical porous structures, it displayed an even higher capacity and energy density [23]. The high gravimetric capacity of 298 F/g was obtained at the discharge current of 1A/g in 1-ethyl-3-methylimidazolium tetrafluoroborate/acetonitrile (EMIMBF_4_/AN) non-aqueous electrolyte, which led to an excellent energy density of 127 Wh/kg. This excellent supercapacitor performance is ascribed to its unique hierarchical porous network that can accommodate relatively large organic electrolyte ions [23].

#### Graphene-based porous composites for supercapacitors

EDLCs generally suffer from limited energy density because their charge storage relies solely on electric double layer capacitive mechanism [102]. On the other hand, pseudocapacitors utilize fast faradaic reactions, and could store the larger amount of charges than EDLCs [98]. Nanostructure hybrid materials composed of graphene and pseudocapacitance materials could be a good platform for high-performance supercapacitors since both electric double layer capacitive and pseudocapacitive mechanisms can be utilized to store charges.

**Fig. 8:**
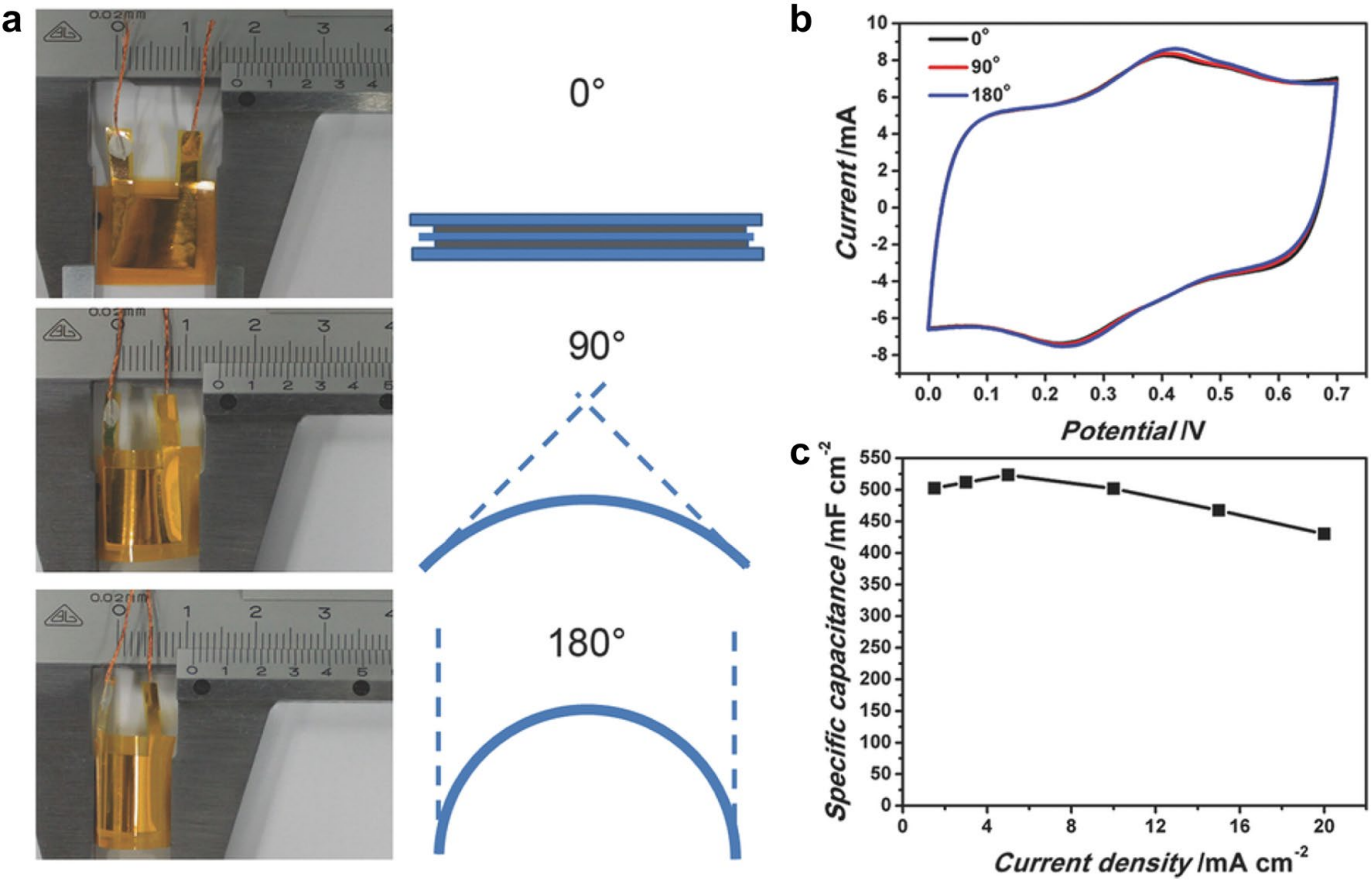
**a** Images and schematics of a flexible film supercapacitor in different bending states. **b** CV curves of the film supercapacitor at scan rates of 20 mV/s when bent by 0°, 90°, 180°. **c** Area-specific capacitance of the flexible film supercapacitor under different current densities (Reprinted with permission [18])

For instance, metal oxide/3D macroporous graphene could store large amount of charges because of its additional pseudocapacitve redox reactions of metal oxides with advantageous macroporous structures. The 3D MnO_2_/macroporous graphene frameworks prepared by template-assisted assembly exhibited an excellent capacitance of 389 F/g at 1 A/g in 1 M Na_2_SO_4_ aqueous electrolyte while 3D macroporous graphene showed around 202 F/g under the same measurement conditions [43]. These results highlight that incorporating pseudocapacitive materials into macroporous frameworks could significantly enhance electrochemical performance such as capacitance and energy density due to additional faradaic reactions. When macroporous graphene networks were coated with an electroactive conducting polymer PANI, high capacitance of 385 F/g was achieved at 0.5 A/g in 1 M H_2_SO_4_ electrolyte, resulting from faradaic reactions of PANI as well as facilitated electrolytes transport caused by 3D macroporous networks [18]. This PANI/graphene composite showed stable electrical performance even in bending states due to superior mechanical properties of graphene, Fig. 8. 3D crumpled graphene/carbon nanotube/PANI also exhibited a high capacitance of 295 F/g in KOH electrolyte when electrochemically inactive commercial PVDF was used as a binder [66]. Its capacitance could be further increased up to 459 F/g by replacing the PVDF binder with graphene sheets [66]. The corresponding energy density was as high as 63.3 Wh/kg [66].

### Lithium-ion batteries

Lithium-ion batteries are the most widely used type of battery ranging from handheld devices to electric vehicles due to their relatively high energy density and stable performance [109]. LIBs are generally composed of a cathode (LiCoO_2_), and an anode (graphite), and non-aqueous electrolyte [99, 109, 110]. Energy is stored based on the intercalation of lithium ions in LIBs where lithium ions move from anodes to cathodes during a discharge process [110]. Energy density, power density, cyclability, safety, rate capability and cost are some of the most important parameters in LIBs. These electrochemical properties are highly dependent on the cathode and anode materials, and can be further improved by engineering of electrode materials [111–123]. Graphene has been used both as an anode material and as an additive to the cathode to enhance the performance of LIBs [114, 117–121, 123].

#### Porous graphene anodes

Graphite is the most widely used anode material in LIBs because of its good reversibility and stability [99, 109, 110]. In graphite anodes, maximum one Li atom can be stored per six C atoms (LiC_6_) based on the intercalation of Li, which gives a theoretical maximum capacity of 372 mAh/g [124, 125]. In the case of graphene, two Li atoms can be stored per six C (Li_2_C_6_) because both sides of graphene are able to store lithium ions, giving a theoretical capacity of 744 mAh/g [24, 126, 127]. Furthermore, more Li atoms can be intercalated in defective sites and edges of graphene, which could lead to even higher capacity than 744 mAh/g.

Indeed, macroporous graphene assembled by the breath figure method showed an extremely high capacity of 3025 mAh/g at a discharge current of 50 mA/g for the first discharge cycle, and lost about 50% of its capacity from the second cycle [17]. This loss of capacity was ascribed to the solid electrolyte interphase formation as well as irreversible redox reactions of oxygen-containing functional groups of graphene sheets [17]. Afterwards, a capacity of 1150 mAh/g was obtained after 50 cycles for self-assembled macroporous graphene whereas nonporous graphene showed a capacity of 678 mAh/g after 50 cycles [17]. In other studies, mesoporous carbon prepared using a CVD method was demonstrated as an anode in LIBs [122]. Mesoporous graphene possessing a high surface area of 1654 m^2^/g with pores of 3–8 nm also exhibited a high capacity of 1723 mAh/g at 0.1 C as well as high rate capacity [122]. Interestingly, it showed promising cycling stability in that no significant capacity decrease was observed [122].

In order to further improve the performance of porous graphene anode, heteroatom doping has been demonstrated. For example, N-doped 3D macroporous graphene was prepared through a two-step process and tested as an anode in LIBs [128]. First, GO sheets were assembled with polystyrene spheres to form PS@GO composite, followed by thermal reduction and N-doping with 5% NH_3_ gas at 550 °C [128]. The resulting anode proved to deliver a specific capacity of 1095 mAh/g after 100 cycles at 200 mAh/g with good rate capability [128]. The 3D structure is beneficial for shortening the diffusion distance of electrons and Li ions leading to higher conductivity, as well as favoring ion migration by allowing easy access of the graphene surface to the electrolyte [128]. Phosphorous and nitrogen dual-doped mesoporous graphene was also demonstrated for LIBs anodes. It was successfully synthesized through a CVD method with a pore-forming template (MgO), carbon, nitrogen, and phosporous sources (CH_4_ and (NH_4_)_3_PO_4_) [123]. It was shown that 0.6 and 2.6 at % of P and N was doped in porous graphene, exhibiting even higher capacity up to 2250 mAh/g at 50 mA/g [123]. This might be due to its defect sites on the graphene sheets caused by P and N doping as well as the porous structure, which allowed for greater interaction with Li^+^ [123]. It also showed a great capacity of 750 mAh/g at a fast discharge current of 1 A/g, which is indicative of its superior rate capability [123].

#### Graphene-based porous cathodes

Current LIBs mostly adopt inorganic cathode materials such as LiCoO_2_, LiFePO_4_, and LiMn_2_O_4_ because of its relatively high capacity and reliable performance [124]. However, their electrical conductivity was generally very low ranging from 10^−9^ to 10^−3^ S/cm, which leads to inferior rate capability and low power of LIBs [28, 129–132]. Incorporating electrically conductive graphene to inorganic cathodes is a good way to increase the overall electrical conductivity, which could potentially result in enhanced electrochemical performance. When porous graphene/inorganic composites are used, higher rate capability can be expected considering its porous nature and high electrochemically accessible surface area.

A continuous 3D graphene/LiFePO_4_ composite was prepared using a spray-drying method followed by thermal annealing process [133]. This graphene/LiFePO_4_ had a substantial void fraction within the 3D micrometer-sized network, which led to improved rate capability and cycling stability [133]. The capacity of this composite was 149 mAh/g at 0.1 C, and 70 mAh/g at 60 C while conventional carbon-coated LiFePO_4_ could deliver only 54 mAh/g at 30 C [133]. This enhanced cathode performance was attributed to its 3D conductive networks as well as shorter diffusion length of Li^+^ caused by its porous structure [133]. Micro- and mesoporous activated graphene/LiFePO_4_ was also demonstrated as a cathode in LIBs [134]. It turned out that Li^+^ ion can migrate much faster in activated graphene/LiFePO_4_ than in LiFePO_4_ and non-activated graphene/LiFePO_4_ because of additional small pores on activated graphene sheets [134]. This activated graphene/LiFePO_4_ showed impressive rate capabilities. At a low discharge rate (20 mA/g), activated graphene/LiFePO_4_, non-activated graphene, and LiFePO_4_ all showed similar capacities around 145 mAh/g [134]. On the other hand, at a high current rate or 5000 mA/g, activated graphene/LiFePO_4_ composites could deliver significantly higher capacity of 60 mAh/g whereas both graphene/LiFePO_4_ and LiFePO_4_ exhibited very low capacities less than 5 mAh/g at such a high current rate [134]. A similar study was performed, in which activated graphene was decorated with 5 nm LiFePO_4_ for a cathode in LIBs [135]. Similarly, it also displayed greater rate capability and cycling stability due mainly to the enhanced electrical conductivity and the improved electrode–electrolyte contacts [135].

In other literatures, redox-active graphene electrodes and its composites have also been demonstrated for cathode materials in LIBs [78, 136–138]. In this strategy, oxygen-containing functional groups on graphene sheets can act as redox centers at ~ 3 V (Vs. Li), which provides pseudocapacitance in addition to electric double layer capacitance [78, 136–138]. For instance, when GO was reduced by tetrahydroxyl-1,4-benzoquinone (THQ) at 80 °C, a 3D functionalized graphene structure was produced, which has a broad pore size distribution from tens of nanometers to tens of micrometers [138]. When used as a cathode, it showed high gravimetric capacities about 165 mAh/g with great cycling stability because of its oxygen-containing functional groups in graphene sheets as well as THQ [138]. Folded graphene sheets produced by hydrothermal reduction of graphene followed by compression and vacuum-drying process also showed a promising gravimetric capacity of ~ 160 mAh/g based on redox-active oxygen-containing functional groups as well as its microstructure [136]. Porous crumpled graphene/few-walled carbon nanotubes composites were also demonstrated as cathode materials, which showed similarly good cathode performance up to ~ 170 mAh/g [78]. The important feature of this type of approach is to effectively utilize the redox-active functional groups on graphene sheet surface by rationally designing conductive 3D porous structures.

### Lithium–sulfur batteries and lithium–air batteries

A great amount of effort has been made to develop more advanced energy storage systems, which could deliver higher energy than currently used commercial LIBs. Li–S and Li–air batteries are among those energy storage systems, which have much higher theoretical capacity and energy density [99]. These batteries have been demonstrated with nanostructured graphene to fully utilize the excellent properties of graphene and enhance its electrochemical performance.

**Fig. 9:**
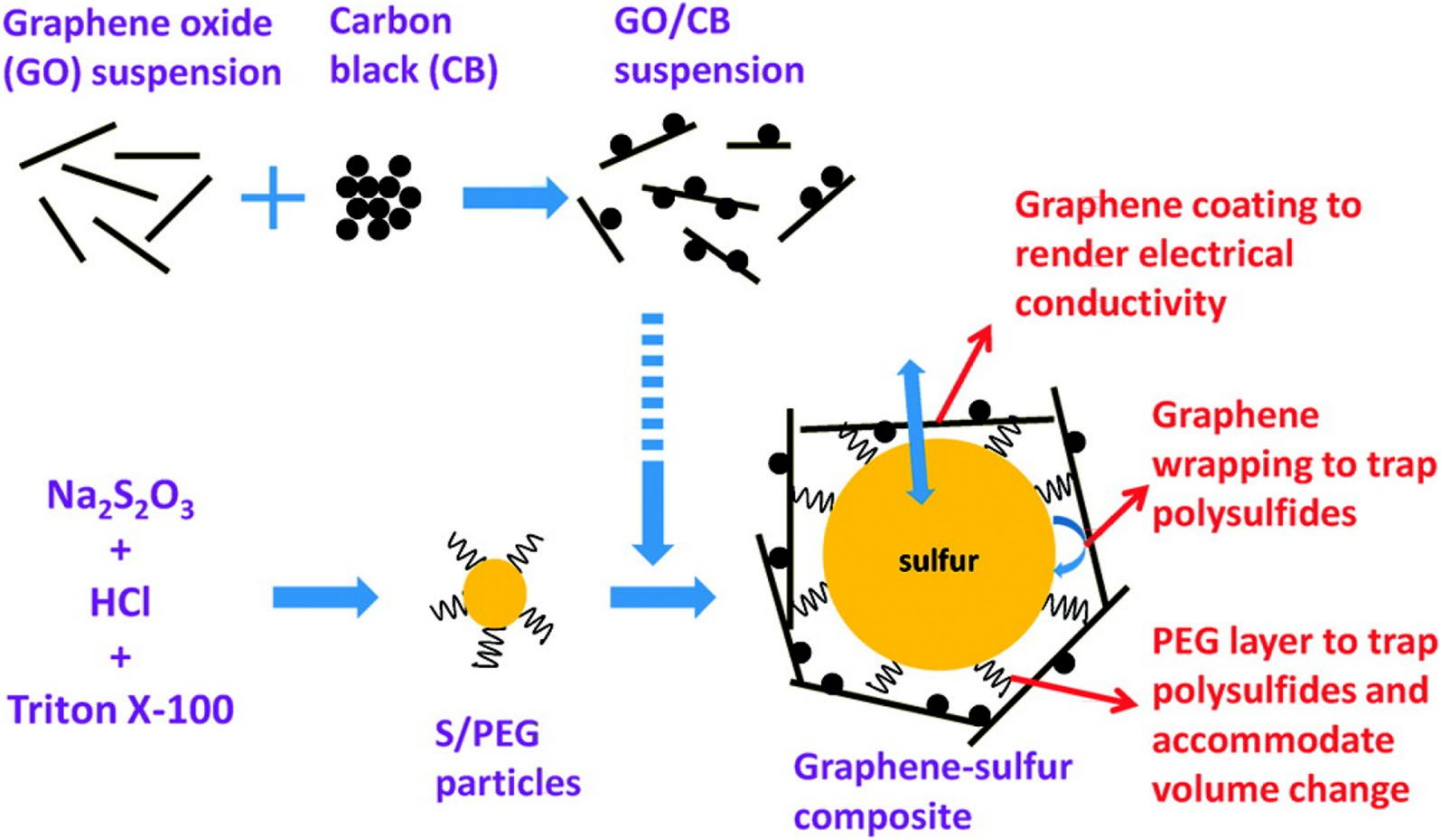
Schematic of the synthesis steps for a graphene-sulfur composite, with a proposed schematic structure of the composite (Reprinted with permission [19])

Li–S batteries have a theoretical capacity of 1675 mAh/g and its theoretical energy density reaches 2600 Wh/kg, which are dramatically higher than those of current LIBs [139]. Despite of its high theoretical capacity, Li–S batteries has not yet successfully replaced LIBs due mainly to its poor electrical conductivity and low cycling stability [140, 141]. During charge and discharge cycles, polysulfides (Li_2_S_x_) are formed and dissolved to electrolytes, leading to a loss in capacity and energy density [140, 141]. Several approaches have been taken to mitigate the shuttle effect of Li–S batteries using porous graphene [19, 142, 143]. Graphene-wrapped sulfur composites were demonstrated, in which carbon black-loaded graphene was assembled with poly(ehtylene glycol) coated sulfur particles, Fig. 9. This graphene-encapsulated sulfur cathodes showed better electrochemical capabilities by effectively reducing the shuttle effect and increasing conductivity, reaching capacity values around 750 mAh/g at 0.2 C for the first cycle [19]. The relatively high capacity around 600 mAh/g was obtained even after 100 cycles [19]. Interestingly, it showed better cycling stability at higher discharge current of 0.5 C probably because it does not enough time for polysulfide shuttle [19]. A hybrid of CNTs and porous graphene was also created, allowing for excellent electrical conductivity, mechanical robustness and interconnected micro and mesopores that favored the accommodation of polysulfides [144]. When used as a cathode material, it showed a high capacity of 1121 mAh g^−1^ at 0.5 C and an excellent rate capability showing 809 mAh g^−1^ at 10 C [144]. Recently, nitrogen-doping has also been utilized in Li–S batteries by synthesizing crumpled, highly porous N-doped graphene sheets with pore volume of 5.4 cm^3^/g, which could accommodate high sulfur content up to 90 wt% in the composite [145]. A high specific capacity was obtained (1486 mAh/g at 0.05 C) with excellent cycling performance (80% retention after 200 cycles) [145]. Nitrogen-doping allowed for a stronger polysulfide absorption in the interwoven N-doped graphene sheets because of the enhanced interaction between nitrogen heteroatoms and polysulfides [145].

Li-air batteries have also great potential because of extremely high theoretical energy density of ~ 11,680 Wh/kg and practical energy density of ~ 3500 Wh/kg, which is even comparable to those of gasoline (theoretical energy density of ~ 13,000 Wh/kg and practical energy density ~ 1700 Wh/kg) [146]. When discharged, oxygen reduction reactions occur at porous cathode leading to the formation of Li_2_O_2_ [147, 148]. At the same time, Li metal anode is oxidized, giving off electrons during discharge processes [148]. Electrochemical performance are dependent on various factors such as composition, conductivity, surface area, porosity of a cathode as well as electrolyte composition [147, 148]. Research efforts in this area have focused on designing porous cathode materials to improve its poor cycling stability and while maintaining high energy density. It was reported that hierarchically assembled porous graphene sheets deliver a very high capacity of 15,000 mAh/g based on the mass of porous graphene without an additional catalyst in pure oxygen [20]. This high capacity is attributed to the unique morphology with the pores providing reaction sites for oxygen reduction reactions and the microporous channels facilitating oxygen diffusion [20]. Defective sites of graphene sheets was proven to suppress the aggregation of Li_2_O_2_ because of strong interaction between defect sites and Li_2_O_2_ [20]. In another paper, ruthenium (Ru) nanocrystal-decorated porous graphene was demonstrated as a cathode for Li-air batteries [149]. It confirmed that pore size and architectures largely influence its electrochemical performance by the fact that nonporous graphene counterpart showed much smaller capacity at identical test conditions [149]. After loading the Ru catalyst, Ru/porous graphene composites exhibited a high capacity of 17,710 at 200 mA/g with a significantly decreased overpotential [149]. When curtailing its capacity to 500 mAh/g during charge/discharge processes, it showed very stable cycling performance with high energy efficiency around 80% [149]. Other graphene-based nanocomposites have been proposed and proved to be effective [150, 151]. For instance, macroporous graphene/graphitic carbon nitride prepared by hydrothermal reduction and freeze-drying exhibited improved capacity higher than 17,000 mAh/g as compared to pristine graphene showing a capacity of 13,000 mAh/g because of synergistic effect of graphene and carbon nitride [151].

## Conclusion and perspective

As highlighted above, porous graphene has been a subject to extensive research and development in the past years due to its unique properties such as excellent mechanical strength and good electrical conductivity, which makes it a promising material in many applications, especially energy storage. This review paper has introduced various synthesis and processing approaches to preparing porous graphene and porous graphene-based nanocomposites with emphasis on energy storage applications. Overall, porous graphene could result in improved electrochemical performance in energy storage systems because of more available active sites and facilitated ion diffusion throughout the porous structures. However, several issues should be addressed to make porous graphene-based energy storage systems commercially viable. First, the porous architecture of graphene should precisely be controlled during preparation to maximize its electrochemical performance. Morphology and pore size along with a graphene wall thickness affect the behavior and electrochemical properties of graphene. Therefore, it is critical to design hierarchically porous structures with optimized pore size and pore volume for achieving superior performance. Second, production process of porous graphene would essentially be environmentally friendly, simple, affordable and scalable. The advancement of these processes will make graphene more readily available for use in high-performance energy storage devices.

Based on the significant effort on porous graphene, it is likely that porous graphene will play important roles in the next-generation energy storage devices. The utilization of porous graphene in supercapacitors, LIBs, Li–S, Li-air and fuel cells will promote manufacturing and commercializing efficient, safe, and more stable energy storage systems. Furthermore, excellent mechanical properties and stability of graphene will provide other opportunities to create multifunctional flexible energy storage devices, which can meet multiple market’s needs simultaneously.

## Abbreviations

AN: acetonitrile
ANF: aramid nanofiber
ATRP: atom transfer radical polymerization
BMIMBF4: 1-butyl-3-methylimidazolium tetrafluoroborate
CCP: crumpled graphene/carbon nanotube/polyaniline
CMG: chemically modified graphene
CNT: carbon nanotube
CRG: chemically reduced graphene oxide
CVD: chemical vapor deposition
DODA-Br: dimethyldioctadecylammonium bromide
EDLC: electric double layer capacitor
EMIMBF4: 1-ethyl-3-methylimidazolium tetrafluoroborate
GO: graphene oxide
IUPAC: International Union of Pure and Applied Chemistry
LbL: layer-by-layer
LIB: lithium-ion battery
Li–S: lithium sulfur
Li-Air: lithium air
MCS: mesoporous carbon spheres
MWCNT: multi-walled carbon nanotube
ORR: oxygen reduction reaction
PANI: polyaniline
PS: polystyrene
PVDF: polyvinylidene fluoride
RGO: reduced graphene oxide
SEI: solid electrolyte interphase
THQ: tetrahydroxyl-1,4-benzoquinone

## References

[1] Allen MJ, Tung VC, Kaner RB (2010). Honeycomb carbon: a review of graphene. Chem. Rev..

[2] Rao CNR, Sood AK, Subrahmanyam KS, Govindaraj A (2009). Graphene: the new two-dimensional nanomaterial. Angew. Chem. Int. Edit..

[3] Novoselov KS, Geim AK, Morozov SV, Jiang D, Zhang Y, Dubonos SV, Grigorieva IV, Firsov AA (2004). Electric field effect in atomically thin carbon films. Science.

[4] Lee C, Wei X, Kysar JW, Hone J (2008). Measurement of the elastic properties and intrinsic strength of monolayer graphene. Science.

[5] Vadukumpully S, Paul J, Mahanta N, Valiyaveettil S (2011). Flexible conductive graphene/poly(vinyl chloride) composite thin films with high mechanical strength and thermal stability. Carbon.

[6] Renteria J, Nika D, Balandin A (2014). Graphene thermal properties: applications in thermal management and energy storage. Appl. Sci..

[7] Balandin AA, Ghosh S, Bao W, Calizo I, Teweldebrhan D, Miao F, Lau CN (2008). Superior thermal conductivity of single-layer graphene. Nano Lett..

[8] Cai W, Moore AL, Zhu Y, Li X, Chen S, Shi L, Ruoff RS (2010). Thermal transport in suspended and supported monolayer graphene grown by chemical vapor deposition. Nano Lett..

[9] Shtein M, Nadiv R, Buzaglo M, Regev O (2015). Graphene-based hybrid composites for efficient thermal management of electronic devices. ACS Appl. Mater. Interfaces..

[10] Xin G, Sun H, Hu T, Fard HR, Sun X, Koratkar N, Borca-Tasciuc T, Lian J (2014). Large-area freestanding graphene paper for superior thermal management. Adv. Mater..

[11] Nair RR, Blake P, Grigorenko AN, Novoselov KS, Booth TJ, Stauber T, Peres NMR, Geim AK (2008). Fine structure constant defines visual transparency of graphene. Science.

[12] Mak KF, Ju L, Wang F, Heinz TF (2012). Optical spectroscopy of graphene: from the far infrared to the ultraviolet. Solid State Commun..

[13] Pirruccio G, Martín Moreno L, Lozano G, Gómez Rivas J (2013). Coherent and broadband enhanced optical absorption in graphene. ACS Nano.

[14] Ye Q, Wang J, Liu Z, Deng Z-C, Kong X-T, Xing F, Chen X-D, Zhou W-Y, Zhang C-P, Tian J-G (2013). Polarization-dependent optical absorption of graphene under total internal reflection. Appl. Phys. Lett..

[15] Li X, Chen W, Zhang S, Wu Z, Wang P, Xu Z, Chen H, Yin W, Zhong H, Lin S (2015). 18.5% efficient graphene/GaAs van der Waals heterostructure solar cell. Nano Energy.

[16] Chen Y, Long Y, Liu Y, Shen L, Zhang Y, Zheng Z, Yu W, Run S (2013). Optimizing the light absorption of graphene-based organic solar cells by tailoring the weak microcavity with dielectric/graphene/dielectric multilayer. Appl. Phys. Lett..

[17] Yin S, Zhang Y, Kong J, Zou C, Li CM, Lu X, Ma J, Boey FYC, Chen X (2011). Assembly of Graphene Sheets into Hierarchical Structures for High-Performance Energy Storage. ACS Nano.

[18] Meng Y, Wang K, Zhang Y, Wei Z (2013). Hierarchical porous graphene/polyaniline composite film with superior rate performance for flexible supercapacitors. Adv. Mater..

[19] Wang H, Yang Y, Liang Y, Robinson JT, Li Y, Jackson A, Cui Y, Dai H (2011). Graphene-wrapped sulfur particles as a rechargeable lithium-sulfur battery cathode material with high capacity and cycling stability. Nano Lett..

[20] Xiao J, Mei D, Li X, Xu W, Wang D, Graff GL, Bennett WD, Nie Z, Saraf LV, Aksay IA, Liu J, Zhang J-G (2011). Hierarchically porous graphene as a lithium-air battery electrode. Nano Lett..

[21] Yun YS, Kim D, Tak Y, Jin H-J (2011). Porous graphene/carbon nanotube composite cathode for proton exchange membrane fuel cell. Synth. Met..

[22] Zhu Y, Murali S, Stoller MD, Ganesh KJ, Cai W, Ferreira PJ, Pirkle A, Wallace RM, Cychosz KA, Thommes M, Su D, Stach EA, Ruoff RS (2011). Carbon-based supercapacitors produced by activation of graphene. Science.

[23] Xu Y, Lin Z, Zhong X, Huang X, Weiss NO, Huang Y, Duan X (2014). Holey graphene frameworks for highly efficient capacitive energy storage. Nat. Commun..

[24] Bonaccorso F, Colombo L, Yu G, Stoller M, Tozzini V, Ferrari AC, Ruoff RS, Pellegrini V (2015). Graphene, related two-dimensional crystals, and hybrid systems for energy conversion and storage. Science.

[25] Choi H-J, Jung S-M, Seo J-M, Chang DW, Dai L, Baek J-B (2012). Graphene for energy conversion and storage in fuel cells and supercapacitors. Nano Energy.

[26] Zhu J, Yang D, Yin Z, Yan Q, Zhang H (2014). Graphene and graphene-based materials for energy storage applications. Small.

[27] Sun Y, Wu Q, Shi G (2011). Graphene based new energy materials. Energy Environ. Sci..

[28] Kucinskis G, Bajars G, Kleperis J (2013). Graphene in lithium ion battery cathode materials: a review. J. Power Sources.

[29] Brownson DAC, Kampouris DK, Banks CE (2011). An overview of graphene in energy production and storage applications. J. Power Sources.

[30] Han S, Wu D, Li S, Zhang F, Feng X (2014). Porous graphene materials for advanced electrochemical energy storage and conversion devices. Adv. Mater..

[31] Russo P, Hu A, Compagnini G (2013). Synthesis, properties and potential applications of porous graphene: a review. NanoMicro Lett..

[32] Reina A, Jia X, Ho J, Nezich D, Son H, Bulovic V, Dresselhaus MS, Kong J (2009). Large area, few-layer graphene films on arbitrary substrates by chemical vapor deposition. Nano Lett..

[33] Li X, Cai W, An J, Kim S, Nah J, Yang D, Piner R, Velamakanni A, Jung I, Tutuc E, Banerjee SK, Colombo L, Ruoff RS (2009). Large-area synthesis of high-quality and uniform graphene films on copper foils. Science.

[34] Hummers WS, Offeman RE (1958). Preparation of graphitic oxide. J. Am. Chem. Soc..

[35] Chen J, Li Y, Huang L, Li C, Shi G (2015). High-yield preparation of graphene oxide from small graphite flakes via an improved Hummers method with a simple purification process. Carbon.

[36] Chen J, Yao B, Li C, Shi G (2013). An improved Hummers method for eco-friendly synthesis of graphene oxide. Carbon.

[37] Li D, Muller MB, Gilje S, Kaner RB, Wallace GG (2008). Processable aqueous dispersions of graphene nanosheets. Nat. Nanotechnol..

[38] Zhang Y, Zhang L, Zhou C (2013). Review of chemical vapor deposition of graphene and related applications. Acc. Chem. Res..

[39] Dean CR, Young AF, Meric I, Lee C, Wang L, Sorgenfrei S, Watanabe K, Taniguchi T, Kim P, Shepard KL, Hone J (2010). Boron nitride substrates for high-quality graphene electronics. Nat. Nanotechnol..

[40] Lee SH, Kim HW, Hwang JO, Lee WJ, Kwon J, Bielawski CW, Ruoff RS, Kim SO (2010). Three-dimensional self-assembly of graphene oxide platelets into mechanically flexible macroporous carbon films. Angew. Chem. Int. Ed..

[41] Chen Z, Ren W, Gao L, Liu B, Pei S, Cheng H-M (2011). Three-dimensional flexible and conductive interconnected graphene networks grown by chemical vapour deposition. Nat. Mater..

[42] Xu C, Xu B, Gu Y, Xiong Z, Sun J, Zhao XS (2013). Graphene-based electrodes for electrochemical energy storage. Energy Environ. Sci..

[43] Choi BG, Yang M, Hong WH, Choi JW, Huh YS (2012). 3D macroporous graphene frameworks for supercapacitors with high energy and power densities. ACS Nano.

[44] Huang X, Sun B, Su D, Zhao D, Wang G (2014). Soft-template synthesis of 3D porous graphene foams with tunable architectures for lithium-O2 batteries and oil adsorption applications. J. Mater. Chem. A.

[45] Shi J-L, Peng H-J, Zhu L, Zhu W, Zhang Q (2015). Template growth of porous graphene microspheres on layered double oxide catalysts and their applications in lithium–sulfur batteries. Carbon.

[46] Zhang L, Chen G, Hedhili MN, Zhang H, Wang P (2012). Three-dimensional assemblies of graphene prepared by a novel chemical reduction-induced self-assembly method. Nanoscale.

[47] Chen W, Yan L (2011). In situ self-assembly of mild chemical reduction graphene for three-dimensional architectures. Nanoscale.

[48] Rouquerol J, Avnir D, Fairbridge CW, Everett DH, Haynes JH, Pernicone N, Ramsay JDF, Sing KSW, Unger KK (1994). Recommendations for the characterization of porous solids. Pure Appl. Chem..

[49] Liu Q, Li ZF, Liu YD, Zhang HY, Ren Y, Sun CJ, Lu WQ, Zhou Y, Stanciu L, Stach EA, Xie J (2015). Graphene-modified nanostructured vanadium pentoxide hybrids with extraordinary electrochemical performance for Li-ion batteries. Nat. Commun..

[50] Dou Y, Jin M, Zhou G, Shui L (2015). Breath figure method for construction of honeycomb films. Membranes.

[51] Lei Z, Christov N, Zhao XS (2011). Intercalation of mesoporous carbon spheres between reduced graphene oxide sheets for preparing high-rate supercapacitor electrodes. Energy Environ. Sci..

[52] Jo EH, Chang H, Kim SK, Choi J-H, Park S-R, Lee CM, Jang HD (2016). One-step synthesis of Pt/graphene composites from pt acid dissolved ethanol via microwave plasma spray pyrolysis. Sci. Rep..

[53] Luo J, Jang HD, Huang J (2013). Effect of sheet morphology on the scalability of graphene-based ultracapacitors. ACS Nano.

[54] Zhao X, Hayner CM, Kung MC, Kung HH (2011). Flexible holey graphene paper electrodes with enhanced rate capability for energy storage applications. ACS Nano.

[55] Zuo Z, Kim TY, Kholmanov I, Li H, Chou H, Li Y (2015). Ultra-light hierarchical graphene electrode for binder-free supercapacitors and lithium-ion battery anodes. Small.

[56] Jiang Z-J, Jiang Z (2014). Fabrication of nitrogen-doped holey graphene hollow microspheres and their use as an active electrode material for lithium ion batteries. ACS Appl. Mater. Interfaces..

[57] Du Z, Ai W, Sun C, Zou C, Zhao J, Chen Y, Dong X, Liu J, Sun G, Yu T, Huang W (2016). Engineering the li storage properties of graphene anodes: defect evolution and pore structure regulation. ACS Appl. Mater. Interfaces..

[58] Xu Y, Chen C-Y, Zhao Z, Lin Z, Lee C, Xu X, Wang C, Huang Y, Shakir MI, Duan X (2015). Solution processable holey graphene oxide and its derived macrostructures for high-performance supercapacitors. Nano Lett..

[59] Jeon JW, Sharma R, Meduri P, Arey BW, Schaef HT, Lutkenhaus JL, Lemmon JP, Thallapally PK, Nandasiri MI, McGrail BP, Nune SK (2014). In situ one-step synthesis of hierarchical nitrogen-doped porous carbon for high-performance supercapacitors. ACS Appl. Mater. Interfaces..

[60] Xu J, Lin Y, Connell JW, Dai L (2015). Nitrogen-doped holey graphene as an anode for lithium-ion batteries with high volumetric energy density and long cycle life. Small.

[61] Jiang ZQ, Jiang ZJ, Tian XN, Chen WH (2014). Amine-functionalized holey graphene as a highly active metal-free catalyst for the oxygen reduction reaction. J. Mater. Chem. A.

[62] Yu DS, Wei L, Jiang WC, Wang H, Sun B, Zhang Q, Goh KL, Si RM, Chen Y (2013). Nitrogen doped holey graphene as an efficient metal-free multifunctional electrochemical catalyst for hydrazine oxidation and oxygen reduction. Nanoscale.

[63] Jiang ZJ, Jiang ZQ, Chen WH (2014). The role of holes in improving the performance of nitrogen-doped holey graphene as an active electrode material for supercapacitor and oxygen reduction reaction. J. Power Sources.

[64] Luo J, Zhao X, Wu J, Jang HD, Kung HH, Huang J (2012). Crumpled graphene-encapsulated si nanoparticles for lithium ion battery anodes. J. Phys. Chem. Lett..

[65] Jang HD, Kim SK, Chang H, Choi J-W, Luo J, Huang J (2013). One-step synthesis of Pt-nanoparticles-laden graphene crumples by aerosol spray pyrolysis and evaluation of their electrocatalytic activity. Aerosol Sci. Technol..

[66] Jo EH, Jang HD, Chang H, Kim SK, Choi J-H, Lee CM (2017). 3 D network-structured crumpled graphene/carbon nanotube/polyaniline composites for supercapacitors. Chemsuschem.

[67] Jeon J-W, Kwon SR, Lutkenhaus JL (2015). Polyaniline nanofiber/electrochemically reduced graphene oxide layer-by-layer electrodes for electrochemical energy storage. J. Mater. Chem. A.

[68] Jang HD, Kim SK, Chang H, Choi J-H, Cho B-G, Jo EH, Choi J-W, Huang J (2015). Three-dimensional crumpled graphene-based platinum–gold alloy nanoparticle composites as superior electrocatalysts for direct methanol fuel cells. Carbon.

[69] Kwon SR, Elinski MB, Batteas JD, Lutkenhaus JL (2017). Robust and flexible aramid nanofiber/graphene layer-by-layer electrodes. ACS Appl. Mater. Interfaces..

[70] Sohn K, Joo Na Y, Chang H, Roh K-M, Dong Jang H, Huang J (2012). Oil absorbing graphene capsules by capillary molding. Chem. Commun..

[71] Huang X, Qian K, Yang J, Zhang J, Li L, Yu C, Zhao D (2012). Functional nanoporous graphene foams with controlled pore sizes. Adv. Mater..

[72] Du J, Lai X, Yang N, Zhai J, Kisailus D, Su F, Wang D, Jiang L (2011). Hierarchically ordered macro—mesoporous TiO2—graphene composite films: improved mass transfer, reduced charge recombination, and their enhanced photocatalytic activities. ACS Nano.

[73] Wen X, Zhang D, Yan T, Zhang J, Shi L (2013). Three-dimensional graphene-based hierarchically porous carbon composites prepared by a dual-template strategy for capacitive deionization. J. Mater. Chem. A.

[74] Cao X, Shi Y, Shi W, Lu G, Huang X, Yan Q, Zhang Q, Zhang H (2011). Preparation of novel 3D graphene networks for supercapacitor applications. Small.

[75] Yu G, Hu L, Vosgueritchian M, Wang H, Xie X, McDonough JR, Cui X, Cui Y, Bao Z (2011). Solution-processed graphene/MnO2 nanostructured textiles for high-performance electrochemical capacitors. Nano Lett..

[76] Ling Z, Wang G, Dong Q, Qian BQ, Zhang MD, Li CP, Qiu JS (2014). An ionic liquid template approach to graphene-carbon xerogel composites for supercapacitors with enhanced performance. J. Mater. Chem. A.

[77] Luo J, Jang HD, Sun T, Xiao L, He Z, Katsoulidis AP, Kanatzidis MG, Gibson JM, Huang J (2011). Compression and aggregation-resistant particles of crumpled soft sheets. ACS Nano.

[78] Lee B, Lee C, Liu T, Eom K, Chen Z, Noda S, Fuller TF, Jang HD, Lee SW (2016). Hierarchical networks of redox-active reduced crumpled graphene oxide and functionalized few-walled carbon nanotubes for rapid electrochemical energy storage. Nanoscale.

[79] Decher G (1997). Fuzzy nanoassemblies: toward layered polymeric multicomposites. Science.

[80] Tang ZY, Wang Y, Podsiadlo P, Kotov NA (2006). Biomedical applications of layer-by-layer assembly: from biomimetics to tissue engineering. Adv. Mater..

[81] Wang Y, Angelatos AS, Caruso F (2008). Template synthesis of nanostructured materials via layer-by-layer assembly. Chem. Mater..

[82] Jiang CY, Tsukruk VV (2006). Freestanding nanostructures via layer-by-layer assembly. Adv. Mater..

[83] Sukhishvili SA (2005). Responsive polymer films and capsules via layer-by-layer assembly. Curr. Opin. Colloid Interface Sci..

[84] Cho C, Wallace KL, Hagen DA, Stevens B, Regev O, Grunlan JC (2015). Nanobrick wall multilayer thin films grown faster and stronger using electrophoretic deposition. Nanotechnology.

[85] Vidyasagar A, Sung C, Gamble R, Lutkenhaus JL (2012). Thermal transitions in dry and hydrated layer-by-layer assemblies exhibiting linear and exponential growth. ACS Nano.

[86] Ariga K, Hill JP, Ji Q (2007). Layer-by-layer assembly as a versatile bottom-up nanofabrication technique for exploratory research and realistic application. Phys. Chem. Chem. Phys..

[87] Hyder MN, Kavian R, Sultana Z, Saetia K, Chen P-Y, Lee SW, Shao-Horn Y, Hammond PT (2014). Vacuum-assisted layer-by-layer nanocomposites for self-standing 3D mesoporous electrodes. Chem. Mater..

[88] Kharlampieva E, Kozlovskaya V, Chan J, Ankner JF, Tsukruk VV (2009). Spin-assisted layer-by-layer assembly: variation of stratification as studied with neutron reflectivity. Langmuir.

[89] Krogman KC, Lowery JL, Zacharia NS, Rutledge GC, Hammond PT (2009). Spraying asymmetry into functional membranes layer-by-layer. Nat. Mater..

[90] Lee SW, Yabuuchi N, Gallant BM, Chen S, Kim B-S, Hammond PT, Shao-Horn Y (2010). High-power lithium batteries from functionalized carbon-nanotube electrodes. Nat. Nano.

[91] Lee SW, Kim B-S, Chen S, Shao-Horn Y, Hammond PT (2009). Layer-by-layer assembly of all carbon nanotube ultrathin films for electrochemical applications. J. Am. Chem. Soc..

[92] Lee SW, Kim J, Chen S, Hammond PT, Shao-Horn Y (2010). Carbon nanotube/manganese oxide ultrathin film electrodes for electrochemical capacitors. ACS Nano.

[93] Lee SW, Gallant BM, Byon HR, Hammond PT, Shao-Horn Y (2011). Nanostructured carbon-based electrodes: bridging the gap between thin-film lithium-ion batteries and electrochemical capacitors. Energy Environ. Sci..

[94] Hyder MN, Lee SW, Cebeci FÇ, Schmidt DJ, Shao-Horn Y, Hammond PT (2011). Layer-by-layer assembled polyaniline nanofiber/multiwall carbon nanotube thin film electrodes for high-power and high-energy storage applications. ACS Nano.

[95] Byon HR, Lee SW, Chen S, Hammond PT, Shao-Horn Y (2011). Thin films of carbon nanotubes and chemically reduced graphenes for electrochemical micro-capacitors. Carbon.

[96] Kwon SR, Jeon J-W, Lutkenhaus JL (2015). Sprayable, paintable layer-by-layer polyaniline nanofiber/graphene electrodes. RSC Adv..

[97] Chen H, Müller MB, Gilmore KJ, Wallace GG, Li D (2008). Mechanically strong, electrically conductive, and biocompatible graphene paper. Adv. Mater..

[98] Simon P, Gogotsi Y (2008). Materials for electrochemical capacitors. Nat. Mater..

[99] Bruce PG, Freunberger SA, Hardwick LJ, Tarascon J-M (2012). Li-O2 and Li-S batteries with high energy storage. Nat. Mater..

[100] Zhang LL, Zhao XS (2009). Carbon-based materials as supercapacitor electrodes. Chem. Soc. Rev..

[101] Wu X-L, Xu A-W (2014). Carbonaceous hydrogels and aerogels for supercapacitors. J. Mater. Chem. A.

[102] Wang G, Zhang L, Zhang J (2012). A review of electrode materials for electrochemical supercapacitors. Chem. Soc. Rev..

[103] Frackowiak E (2007). Carbon materials for supercapacitor application. Phys. Chem. Chem. Phys..

[104] Zhang LL, Zhou R, Zhao XS (2010). Graphene-based materials as supercapacitor electrodes. J. Mater. Chem..

[105] Xu Y, Sheng K, Li C, Shi G (2010). Self-assembled graphene hydrogel via a one-step hydrothermal process. ACS Nano.

[106] Stoller MD, Park S, Zhu Y, An J, Ruoff RS (2008). Graphene-based ultracapacitors. Nano Lett..

[107] Oh YJ, Yoo JJ, Kim YI, Yoon JK, Yoon HN, Kim J-H, Park SB (2014). Oxygen functional groups and electrochemical capacitive behavior of incompletely reduced graphene oxides as a thin-film electrode of supercapacitor. Electrochim. Acta.

[108] Suktha P, Chiochan P, Iamprasertkun P, Wutthiprom J, Phattharasupakun N, Suksomboon M, Kaewsongpol T, Sirisinudomkit P, Pettong T, Sawangphruk M (2015). High-performance supercapacitor of functionalized carbon fiber paper with high surface ionic and bulk electronic conductivity: effect of organic functional groups. Electrochim. Acta.

[109] Tarascon JM, Armand M (2001). Issues and challenges facing rechargeable lithium batteries. Nature.

[110] Manthiram A (2011). Materials challenges and opportunities of lithium ion batteries. J. Phys. Chem. Lett..

[111] Ma Z, Shao G, Fan Y, Wang G, Song J, Liu T (2014). Tunable morphology synthesis of LiFePO4 nanoparticles as cathode materials for lithium ion batteries. ACS Appl. Mater. Interfaces..

[112] Marzec J, Świerczek K, Przewoźnik J, Molenda J, Simon DR, Kelder EM, Schoonman J (2002). Conduction mechanism in operating a LiMn2O4 cathode. Solid State Ionics.

[113] Prosini PP, Lisi M, Zane D, Pasquali M (2002). Determination of the chemical diffusion coefficient of lithium in LiFePO4. Solid State Ionics.

[114] Zhao D, Cao M (2015). Constructing highly graphitized carbon-wrapped Li3VO4 nanoparticles with hierarchically porous structure as a long life and high capacity anode for lithium-ion batteries. ACS Appl. Mater. Interfaces..

[115] Iwama E, Kawabata N, Nishio N, Kisu K, Miyamoto J, Naoi W, Rozier P, Simon P, Naoi K (2016). enhanced electrochemical performance of ultracentrifugation-derived nc-Li3VO4/MWCNT composites for hybrid supercapacitors. ACS Nano.

[116] Shao G, Gan L, Ma Y, Li H, Zhai T (2015). Enhancing the performance of Li3VO4 by combining nanotechnology and surface carbon coating for lithium ion batteries. J. Mater. Chem. A.

[117] Liu J, Lu P-J, Liang S, Liu J, Wang W, Lei M, Tang S, Yang Q (2015). Ultrathin Li3VO4 nanoribbon/graphene sandwich-like nanostructures with ultrahigh lithium ion storage properties. Nano Energy.

[118] Zhao X, Hayner CM, Kung HH (2011). Self-assembled lithium manganese oxide nanoparticles on carbon nanotube or graphene as high-performance cathode material for lithium-ion batteries. J. Mater. Chem..

[119] Sreelakshmi KV, Sasi S, Balakrishnan A, Sivakumar N, Nair AS, Nair SV, Subramanian KRV (2014). Hybrid composites of LiMn2O4—graphene as rechargeable electrodes in energy storage devices. Energy Technol..

[120] Fathollahi F, Javanbakht M, Omidvar H, Ghaemi M (2015). Improved electrochemical properties of LiFePO4/graphene cathode nanocomposite prepared by one-step hydrothermal method. J. Alloy. Compd..

[121] Guo X, Fan Q, Yu L, Liang J, Ji W, Peng L, Guo X, Ding W, Chen Y (2013). Sandwich-like LiFePO4/graphene hybrid nanosheets: in situ catalytic graphitization and their high-rate performance for lithium ion batteries. J. Mater. Chem. A.

[122] Fan Z, Yan J, Ning G, Wei T, Zhi L, Wei F (2013). Porous graphene networks as high performance anode materials for lithium ion batteries. Carbon.

[123] Ma X, Ning G, Qi C, Xu C, Gao J (2014). Phosphorus and nitrogen dual-doped few-layered porous graphene: a high-performance anode material for lithium-ion batteries. ACS Appl. Mater. Interfaces.

[124] Nitta N, Wu F, Lee JT, Yushin G (2015). Li-ion battery materials: present and future. Mater. Today.

[125] Campbell B, Ionescu R, Tolchin M, Ahmed K, Favors Z, Bozhilov KN, Ozkan CS, Ozkan M (2016). Carbon-coated, diatomite-derived nanosilicon as a high rate capable Li-ion battery anode. Sci. Rep..

[126] Wang G, Shen X, Yao J, Park J (2009). Graphene nanosheets for enhanced lithium storage in lithium ion batteries. Carbon.

[127] Dahn JR, Zheng T, Liu Y, Xue JS (1995). Mechanisms for lithium insertion in carbonaceous materials. Science.

[128] Liu X, Wu Y, Yang Z, Pan F, Zhong X, Wang J, Gu L, Yu Y (2015). Nitrogen-doped 3D macroporous graphene frameworks as anode for high performance lithium-ion batteries. J. Power Sources.

[129] Kim D-K, Park H-M, Jung S-J, Jeong YU, Lee J-H, Kim J-J (2006). Effect of synthesis conditions on the properties of LiFePO4 for secondary lithium batteries. J. Power Sources.

[130] Molenda J, Ojczyk W, Marzec M, Marzec J, Przewoźnik J, Dziembaj R, Molenda M (2003). Electrochemical and chemical deintercalation of LiMn2O4. Solid State Ionics.

[131] Park M, Zhang X, Chung M, Less GB, Sastry AM (2010). A review of conduction phenomena in Li-ion batteries. J. Power Sources.

[132] Levasseur S, Ménétrier M, Delmas C (2002). On the dual effect of Mg doping in LiCoO2 and Li1+ δCoO2: structural, electronic properties, and 7Li MAS NMR studies. Chem. Mater..

[133] Zhou X, Wang F, Zhu Y, Liu Z (2011). Graphene modified LiFePO4 cathode materials for high power lithium ion batteries. J. Mater. Chem..

[134] Ha J, Park S-K, Yu S-H, Jin A, Jang B, Bong S, Kim I, Sung Y-E, Piao Y (2013). A chemically activated graphene-encapsulated LiFePO4 composite for high-performance lithium ion batteries. Nanoscale.

[135] Dutta D, Santhosha AL, Sood AK, Bhattacharyya AJ (2016). Reducing Li-diffusion pathways via “adherence” of ultra-small nanocrystals of LiFePO4 on few-layer nanoporous holey-graphene sheets for achieving high rate capability. RSC Adv..

[136] Liu T, Kim KC, Kavian R, Jang SS, Lee SW (2015). High-density lithium-ion energy storage utilizing the surface redox reactions in folded graphene films. Chem. Mater..

[137] Ha SH, Jeong YS, Lee YJ (2013). Free standing reduced graphene oxide film cathodes for lithium ion batteries. ACS Appl. Mater. Interfaces..

[138] Liu T, Kavian R, Kim I, Lee SW (2014). Self-assembled, redox-active graphene electrodes for high-performance energy storage devices. J. Phys. Chem. Lett..

[139] Xu G, Ding B, Pan J, Nie P, Shen L, Zhang X (2014). High performance lithium-sulfur batteries: advances and challenges. J. Mater. Chem. A.

[140] Manthiram A, Fu Y, Su Y-S (2013). Challenges and prospects of lithium-sulfur batteries. Acc. Chem. Res..

[141] Ji X, Nazar LF (2010). Advances in Li-S batteries. J. Mater. Chem..

[142] Wu H, Huang Y, Zong M, Fu H, Sun X (2015). Self-assembled graphene/sulfur composite as high current discharge cathode for lithium-sulfur batteries. Electrochim. Acta.

[143] Zhou G, Yin L-C, Wang D-W, Li L, Pei S, Gentle IR, Li F, Cheng H-M (2013). Fibrous hybrid of graphene and sulfur nanocrystals for high-performance lithium-sulfur batteries. ACS Nano.

[144] Peng HJ, Huang JQ, Zhao MQ, Zhang Q, Cheng XB, Liu XY, Qian WZ, Wei F (2014). Nanoarchitectured graphene/CNT@porous carbon with extraordinary electrical conductivity and interconnected micro/mesopores for lithium-sulfur batteries. Adv. Funct. Mater..

[145] Song J, Yu Z, Gordin ML, Wang D (2016). Advanced sulfur cathode enabled by highly crumpled nitrogen-doped graphene sheets for high-energy-density lithium-sulfur batteries. Nano Lett..

[146] Girishkumar G, McCloskey B, Luntz AC, Swanson S, Wilcke W (2010). Lithium—air battery: promise and challenges. J. Phys. Chem. Lett..

[147] Ma Z, Yuan X, Li L, Ma Z-F, Wilkinson DP, Zhang L, Zhang J (2015). A review of cathode materials and structures for rechargeable lithium-air batteries. Energy Environ. Sci..

[148] Aurbach D, McCloskey BD, Nazar LF, Bruce PG (2016). Advances in understanding mechanisms underpinning lithium–air batteries. Nat. Energy.

[149] Sun B, Huang X, Chen S, Munroe P, Wang G (2014). Porous graphene nanoarchitectures: an efficient catalyst for low charge-overpotential, long life, and high capacity lithium-oxygen batteries. Nano Lett..

[150] Sun C, Li F, Ma C, Wang Y, Ren Y, Yang W, Ma Z, Li J, Chen Y, Kim Y, Chen L (2014). Graphene-Co3O4 nanocomposite as an efficient bifunctional catalyst for lithium-air batteries. J. Mater. Chem. A.

[151] Luo W-B, Chou S-L, Wang J-Z, Zhai Y-C, Liu H-K (2015). A metal-free, free-standing, macroporous graphene@g-C3N4 composite air electrode for high-energy lithium oxygen batteries. Small.

